# Oral Delivery of Mesenchymal Stem Cell-Derived Extracellular Vesicles To Treat Intestinal Inflammation

**DOI:** 10.1021/acsami.6c07496

**Published:** 2026-06-30

**Authors:** Mona Belaid, Wei Heng Chng, Ram Pravin Kumar Muthuramalingam, Yun Wei Lim, Jana Javorovic, Yunyue Zhang, Xiang Luo, Bertrand Czarny, Driton Vllasaliu

**Affiliations:** † Institute of Pharmaceutical Science, 4616 King’s College London, London SE1 9NH, United Kingdom; ‡ Department of Pharmacy and Pharmaceutical Sciences, 37580 National University of Singapore, Singapore 117543, Singapore; § School of Materials Science and Engineering, 54761 Nanyang Technological University, Singapore 639798, Singapore

**Keywords:** mesenchymal stem cell-derived extracellular vesicles, oral delivery, colon targeting, inflammatory bowel disease, colitis

## Abstract

Despite advances in therapy for inflammatory bowel disease (IBD), current treatments are associated with poor clinical outcomes and systemic side effects. Mesenchymal stem cell-derived extracellular vesicles (MSC-EVs) have therapeutic potential in IBD due to their regenerative and immunomodulatory properties. However, most studies administer MSC-EVs by injection, which does not offer the significant benefits of oral administration, including direct and localized access to the site(s) of intestinal inflammation. Here, we evaluated the stability of MSC-EVs for oral delivery by assessing particle size, concentration, and EV markers. MSC-EVs disintegrated in gastrointestinal (GI) fluids, with cryogenic electron microscopy confirming the loss of structural integrity. To address this, we developed a double-coating formulation consisting of chitosan and Eudragit S100 to enhance GI stability and facilitate colon-targeted delivery. Coated EVs were resistant to GI fluids and digestive enzymes, and the formulation released structurally intact, biologically active vesicles in colonic fluid. Preliminary in vivo studies showed that orally administered coated EVs reduced disease severity in a colitis mouse model and elicited a stronger therapeutic response than uncoated EVs administered orally or intravenously at the same dose. These findings indicate that, with appropriate formulation, oral delivery of MSC-EVs could be an effective route of administration to treat intestinal inflammation.

## Graphical abstract

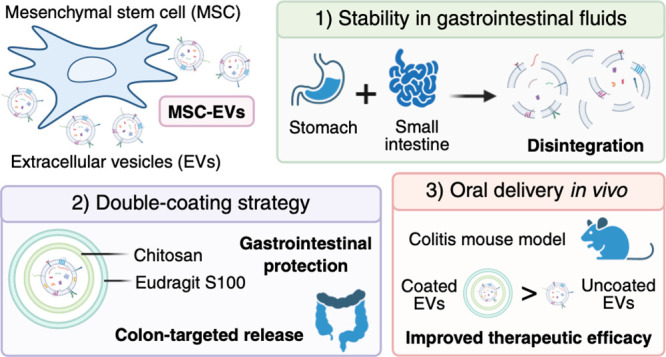

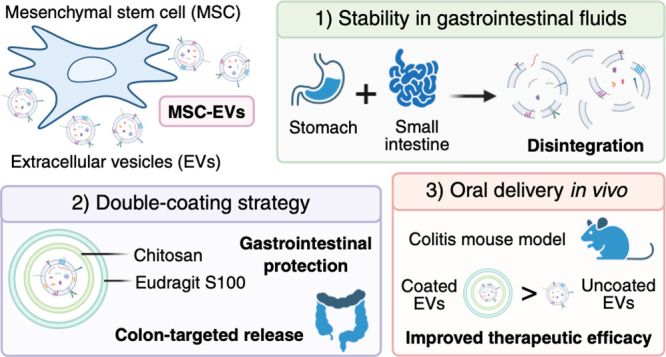

## Introduction

Inflammatory bowel disease (IBD) is a chronic and incurable inflammatory condition of the gastrointestinal tract. Damage to the intestinal mucosa due to infectious agents, toxins, environmental factors or an imbalance in the regulating components of the mucosal barrier can result in dysfunction and loss of barrier integrity.^1,2^ This disruption affects the host-microbial balance, leading to dysbiosis, and activates the immune system, triggering inflammatory signaling cascades that result in intestinal inflammation.^3,4^ The two major classifications of IBD include Crohn’s disease and ulcerative colitis. IBD emerged as a global public health challenge at the turn of the 21st century,^5,6^ with an estimated 7 million people affected worldwide,^7,8^ and its prevalence is expected to continue rising over the next two decades.^9,10^ IBD poses a substantial burden on global healthcare systems and society, resulting in high medical expenses, lost productivity and a reduced quality of life for affected individuals.^5,11,12^ While biological therapies have improved the management of IBD, they are administered systemically by injection and are associated with high cost, toxicity and loss of therapeutic response over time.^13–15^ Despite the availability of advanced treatment options, many individuals with IBD continue to experience suboptimal disease control and substantial gaps remain in the appropriate management of the condition.^16^ There is therefore an unmet need for novel, safe and effective IBD therapies.

Extracellular vesicles (EVs) are cell-derived membrane-bound nanoparticles that mediate intercellular communication, elicit functional responses and promote phenotypic changes that influence the cells’ physiological or pathological status.^17^ Cells release EVs containing membrane and cytosolic components with specific lipid, protein and nucleic acid compositions that reflect their biogenesis and determine their biological role.^18–21^ Mesenchymal stem cell-derived extracellular vesicles (MSC-EVs) have been investigated for their therapeutic potential in IBD owing to their inherent regenerative and immunomodulatory properties. In IBD mouse models, MSC-EVs have been shown to promote mucosal healing and regeneration of the intestinal epithelium, restore barrier integrity, suppress pro-inflammatory cytokine production, reduce macrophage infiltration in colon tissues and promote M2 macrophage polarization, and improve gut microbiota composition.^22–31^ Given the existing literature, this study does not aim to further investigate the mechanisms underlying the therapeutic effects of MSC-EVs in vivo.

To date, current studies with MSC-EVs in IBD animal models mainly use intravenous or intraperitoneal injections for the administration of EVs.^32,33^ Systemically administered EVs are rapidly cleared from the bloodstream, primarily by the mononuclear phagocyte system, limiting their bioavailability and therapeutic efficacy at the site of action.^34^ As a result, systemic administration of EVs often requires higher doses, which can potentially increase the risk of off-target effects as well as treatment costs. Effective treatment of IBD requires targeted delivery of therapeutics to the intestine^35^ and the oral route is considered both the most suitable for achieving localized intestinal delivery and the most convenient for patients.^36^ Oral administration of MSC-EVs would overcome the issues associated with intravenous administration and eliminate the requirement for a trained healthcare professional to administer the therapy.

In this study, we investigated the stability of MSC-EVs in gastrointestinal fluids and developed a coating strategy to enhance their oral stability and enable colon-targeted delivery. We then conducted preliminary in vivo studies to evaluate the therapeutic efficacy of orally administered coated and uncoated MSC-EVs, as well as intravenously administered EVs, in a mouse model of colitis.

## Results and Discussion

### Biophysical and Biochemical Characterization of MSC-EVs

EVs were isolated from the conditioned medium of human bone marrow mesenchymal stem cells. The size distribution and concentration of EVs were measured using nanoparticle tracking analysis (Figure 1a). 90% of isolated EVs were smaller than 200 nm in diameter and most batches had a particle to protein ratio (P:P) > 3 × 10^10^ particles/μg, indicating high vesicular purity^37^ (Figure 1b). The EVs had a mean surface charge of −17 mV as determined by zeta potential measurements. Cryogenic electron microscopy (cryo-EM) showed the morphology of EVs as spherical membrane-bound vesicles (Figure 1c). EV markers were detected using an exosome antibody array and the vesicles showed positive expression for transmembrane proteins CD63 and CD81 and cytosolic proteins TSG101, ALIX and FLOT1, and negative expression for cis-Golgi matrix protein GM130, confirming the absence of cellular contamination in the isolated EV preparation^38,39^ (Figure 1d). Super-resolution microscopy also confirmed the colocalization of tetraspanins CD63, CD81 and CD9 on single EVs (Figure 1e).

**Figure 1:**
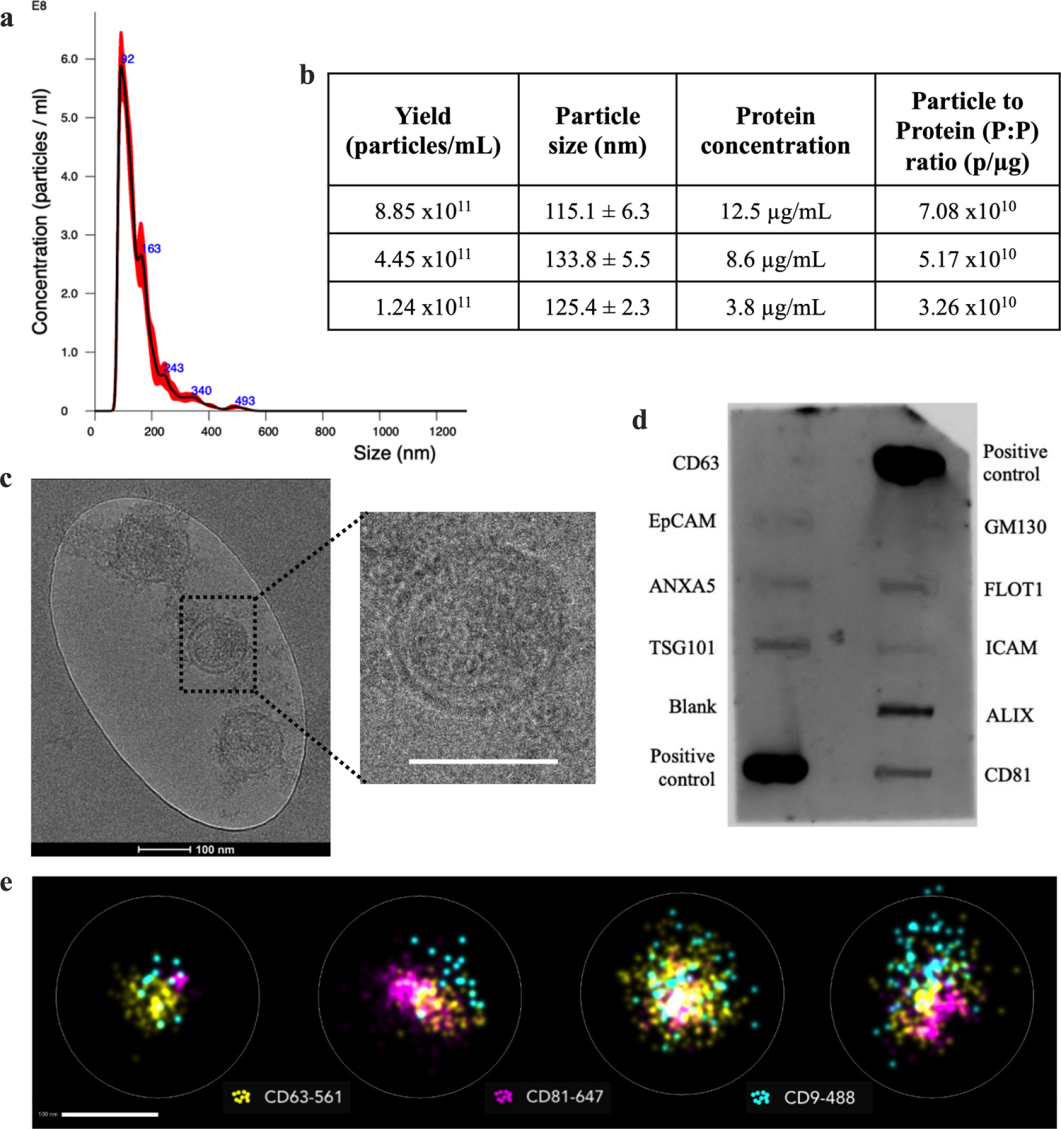
Biophysical and biochemical characterization of MSC-EVs. (a) Size distribution of MSC-EVs using nanoparticle tracking analysis (NTA). The NTA histogram represents repeated captures of a single EV preparation, with the red shaded areas denoting standard deviation between measurements. (b) Table summarizing the characterization of three different EV preparations. Yield and particle size were measured by NTA. Protein concentration was measured using a bicinchoninic acid (BCA) assay. The particle-to-protein (P:P) ratio was calculated using the formula: P:P ratio = yield/protein concentration. (c) Cryogenic electron microscopy (cryo-EM) image of MSC-EVs showing spherical vesicular morphology with a bilayer membrane. Scale bar: 100 nm. (d) Detection of EV-associated proteins using an exosome antibody array. CD63 and CD81: tetraspanins; EpCAM: epithelial cell adhesion molecule; ANXA5: annexin A5; TSG101: tumor susceptibility gene 101; FLOT1: flotillin-1; ICAM: intercellular adhesion molecule 1; and ALIX: programmed cell death 6 interacting protein. The array also included four controls: GM130: cis-Golgi matrix protein as a negative marker for cellular contamination, two positive controls, and one blank. (e) Super-resolution microscopy of MSC-EVs stained for three tetraspanins: CD63 (yellow), CD81 (pink), and CD9 (blue). Scale bar: 100 nm.

### MSC-EVs Possess Regenerative and Immunomodulatory Properties

To assess cellular responses to MSC-EVs, we performed in vitro assays using three concentrations: 10^7^, 10^9^, and 10^11^ particles per mL (p/mL).

We examined the effects of MSC-EVs on metabolic activity and proliferation in Caco-2 epithelial cells. Cells incubated with EVs at 10^7^ and 10^9^ p/mL for 4 days showed a significant increase in cell density, whereas 10^11^ p/mL EVs produced results similar to the control group (Figure 2a). None of the EV concentrations diminished Caco-2 cell viability, unlike the strong cytotoxic effect observed with 10% dimethyl sulfoxide (DMSO). Caco-2 cells seeded in culture inserts with a cell-free gap and incubated with MSC-EVs were imaged every 24 h to monitor wound healing (Figure 2b). Epithelial wound repair requires a combination of cell proliferation and migration.^40^ EVs at 10^9^ p/mL significantly increased the percentage of wound closure at 48 and 72 h (Figure 2c) and the rate of cell migration at 24 h (Figure 2d), outperforming the other EV concentrations. EVs at 10^7^ p/mL also significantly increased wound closure percentage at 72 h but did not affect the rate of Caco-2 cell migration, while 10^11^ p/mL EVs were comparable to the control group.

**Figure 2:**
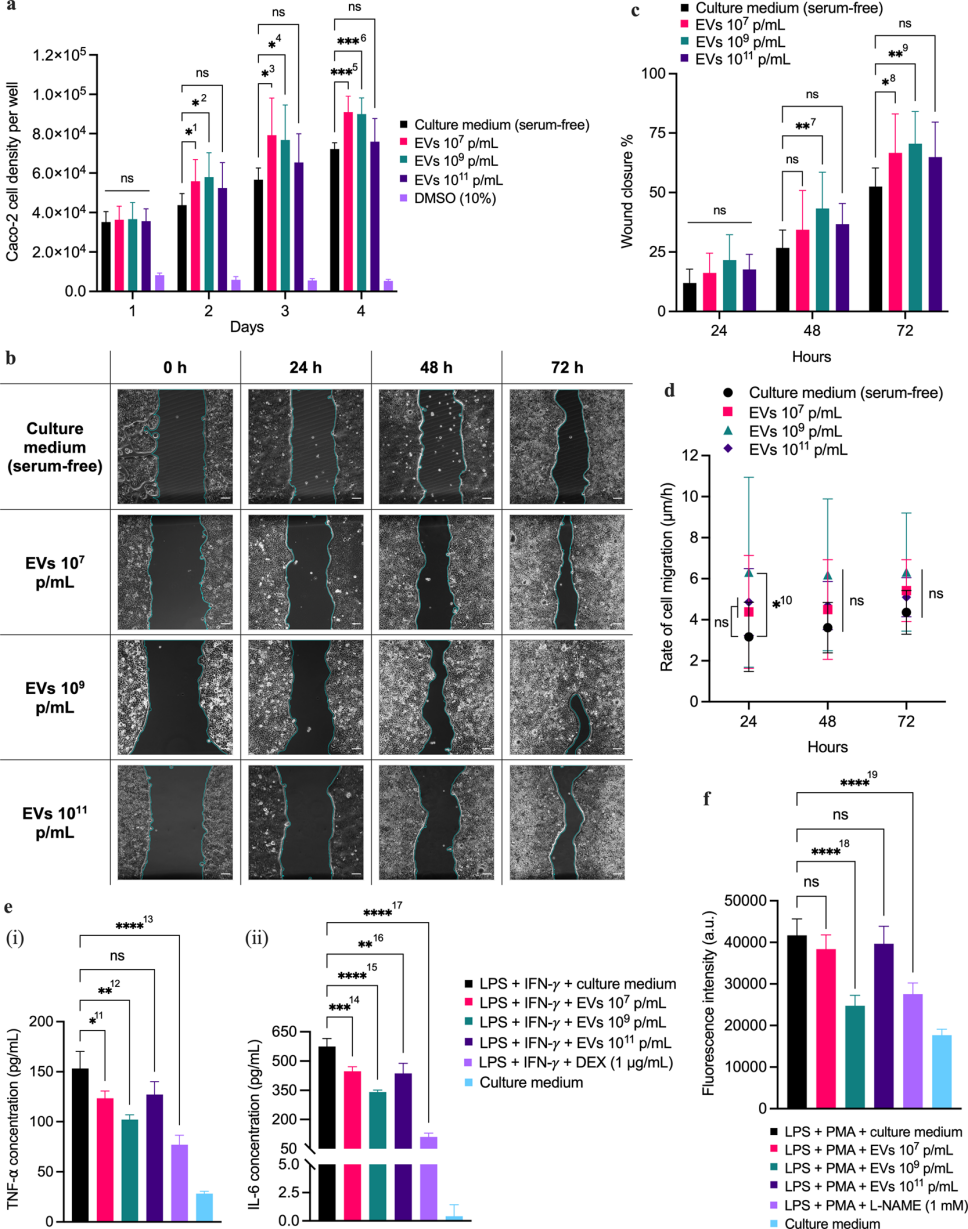
**Regenerative and immunomodulatory properties of MSC-EVs**. Assays were performed with three EV concentrations: 10^7^, 10^9^, and 10^11^ p/mL (a) Effect of MSC-EVs on cell proliferation. Caco-2 cell density per well was obtained using the MTS assay standard curve (Figure S2). EVs at 10^7^ and 10^9^ p/mL significantly increased Caco-2 cell density per well on day 2 (^1^* *p* = 0.0391, ^2^* *p* = 0.0287), day 3 (^3^* *p* = 0.0219, ^4^* *p* = 0.0296), and day 4 (^5^*** *p* = 0.0002, ^6^*** *p* = 0.0004). 10% Dimethyl sulfoxide (DMSO) was used as a negative control for cell proliferation. (b) Wound healing images of Caco-2 cells captured every 24 h and analyzed using Image J to measure wound area and width. Scale bar: 100 μm. (c) Effect of MSC-EVs on wound closure percentage over time. 10^9^ p/mL EVs significantly accelerated Caco-2 wound closure after 48 h (^7^** *p* = 0.0092) and 72 h (^9^** *p* = 0.0040). 10^7^ p/mL EVs showed a significant increase in wound closure after 72 h (^8^* *p* = 0.0309). (d) Effect of MSC-EVs on the rate of cell migration. 10^9^ p/mL EVs significantly increased the rate of Caco-2 cell migration after 24 h (^10^* *p* = 0.0181). (e) Effect of MSC-EVs on cytokine production: (i) TNF-α and (ii) IL-6. J774A.1 macrophages were activated with lipopolysaccharide (LPS) and IFN-γ and cytokine levels were quantified using the Luminex assay. EVs at 10^7^ and 10^9^ p/mL significantly decreased the production of TNF-α (^11^* *p* = 0.0242, ^12^** *p* = 0.0017) and IL-6 (^14^*** *p* = 0.0008, ^15^**** *p* < 0.0001). EVs 10^11^ p/mL did not affect TNF-α but did reduce the concentration of IL-6 (^16^** *p* = 0.0029). One μg/mL Dexamethasone (DEX) was used as a positive control for anti-inflammatory activity and significantly inhibited the production of both TNF-α and IL-6 (^13^**** and ^17^**** *p* < 0.0001). (f) Effect of MSC-EVs on oxidative stress. J774A.1 macrophages were stimulated with LPS and phorbol 12-myristate 13-acetate (PMA), and reactive oxygen species (ROS) were detected by measuring fluorescence intensity following DCFH-DA staining. 10^9^ p/mL was the only EV concentration that significantly decreased ROS production (^18^**** *p* < 0.0001). L-NAME: Nω-Nitro-l-arginine methyl ester. One mmol/L L-NAME was used as a positive control for antioxidant activity and significantly reduced oxidative stress (^19^**** *p* < 0.0001). Data are presented as mean ± SD (*n* = 3). (a), (c), and (d) Two-way ANOVA with posthoc Dunnett’s test. (e) and (f) Brown-Forsythe and Welch ANOVA with posthoc Dunnett’s T3 test. (ns, *p* > 0.05; * *p* < 0.05; ** *p* < 0.01; *** *p* < 0.001; **** *p* < 0.0001).

We evaluated the effects of MSC-EVs on cytokine production and oxidative stress during inflammation in J774A.1 macrophages. Lipopolysaccharide (LPS) stimulation in the presence of IFN-γ polarizes macrophages toward the M1 phenotype, which is characterized by the production of high levels of pro-inflammatory cytokines, such as TNF-α and IL-6.^41^ Activated J774A.1 cells incubated with EVs at 10^7^ and 10^9^ p/mL showed a significant reduction in TNF-α levels after 24 h (Figure 2ei). All three EV concentrations significantly lowered IL-6 levels after 24 h, with 10^9^ p/mL EVs exerting the strongest effect (Figure 2eii). Exposure to LPS and phorbol 12-myristate 13-acetate (PMA) induces cellular oxidative stress, as pathogen-derived compounds increase the production of reactive oxygen and nitrogen species (ROS and RNS, respectively) in macrophages.^42^ Only 10^9^ p/mL EVs significantly reduced ROS production in J774A.1 macrophages after 24 h, whereas 10^7^ and 10^11^ p/mL EVs produced similar outcomes to the control group (Figure 2f).

These results demonstrated that the most effective EV dose in vitro was 10^9^ p/mL. At this concentration, MSC-EVs enhanced metabolic activity, proliferation and wound healing in epithelial cells, while reducing cytokine production and oxidative stress in inflamed macrophages. EVs at 10^7^ p/mL elicited positive effects on cell proliferation, wound healing and pro-inflammatory cytokine release, but did not affect ROS production and were overall less effective than 10^9^ p/mL EVs. By comparison, EVs at 10^11^ p/mL had little impact on cells apart from reducing IL-6 secretion, possibly due to degradation caused by up-regulation of lysosomal activity at high EV doses.^43^

### MSC-EVs Cross Intestinal Epithelial Monolayers, Reduce Inflammation, and Restore Epithelial Barrier Integrity in a Coculture Model of Intestinal Inflammation

We investigated the effect of MSC-EVs in a validated Caco-2/J774A.1 coculture model that mimics intestinal inflammation.^44^ The model consists of human Caco-2 intestinal epithelial cells and murine J774A.1 macrophages cultured in a Transwell system, which comprises a semipermeable membrane insert separating the apical (upper) and basolateral (lower) compartments (Figure 3a). Caco-2 cells were seeded on Transwell inserts and maintained in culture for 19–21 days to allow differentiation into polarized intestinal epithelial monolayers. The transepithelial electrical resistance (TEER) values were monitored throughout the culture period to assess epithelial barrier integrity and tight junction formation in differentiated Caco-2 monolayers.

**Figure 3:**
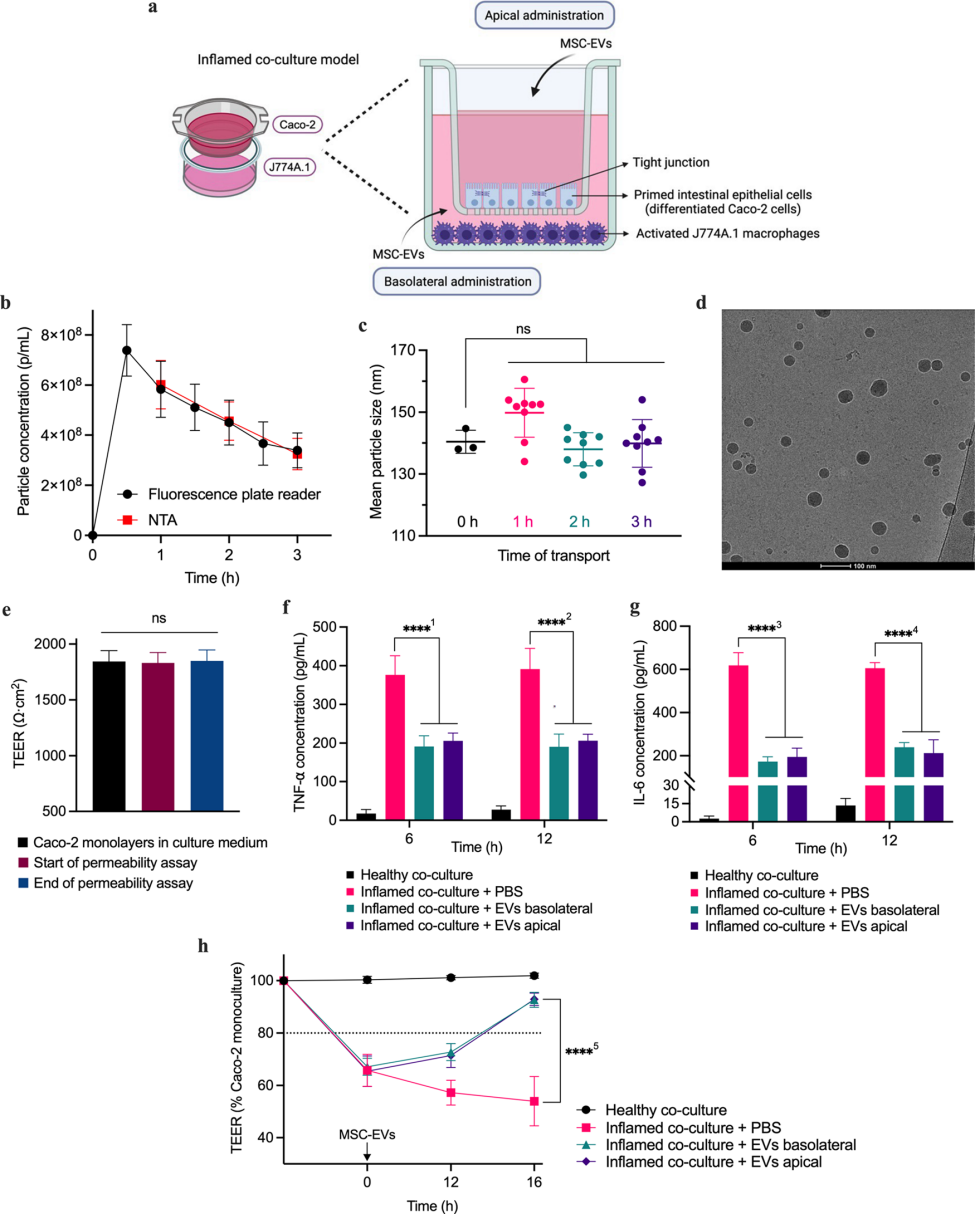
**Transport of MSC-EVs across intestinal epithelial monolayers and their effect in a coculture model of intestinal inflammation.** (a) Schematic representation of the inflamed coculture model. Caco-2 cells were seeded on Transwell inserts and maintained in culture for 19–21 days to allow differentiation into intestinal epithelial monolayers. Differentiated Caco-2 cells were primed with cytokines (TNF-α, IFN-γ, and IL-1β) and J774A.1 macrophages were stimulated with LPS and IFN-γ prior to coculture assembly. MSC-EVs were applied to the coculture either in the apical (upper) or basolateral (lower) compartment. Created with BioRender.com. For transport studies, MSC-EVs (1 × 10^9^ p/mL) labeled with wheat germ agglutinin-Alexa Fluor 488 (WGA-AF488) were applied to the apical compartment of differentiated Caco-2 monolayers in HBSS and incubated at 37 °C for 3 h. (b) Particle concentration in the basolateral compartment of differentiated Caco-2 monolayers over 3 h. Samples were collected every 30 min and analyzed using a fluorescence plate reader and nanoparticle tracking analysis (NTA). EV transport occurred rapidly during the first 30 min of the assay, followed by a gradual reduction in transport rate over time, potentially due to decreasing concentration gradients between the apical and basolateral compartments. Internalization of EVs by differentiated Caco-2 cells may also have contributed to reduced transport (Figure S5). (c) Mean particle size of transported MSC-EVs measured by NTA. Particle size did not change significantly during transport, suggesting that EVs remained structurally intact after permeating the Caco-2 epithelial monolayer (ns, *p* > 0.05). (d) Representative cryogenic electron microscopy (cryo-EM) image of transported EVs showing structurally intact vesicles. Scale bar: 100 nm. (e) Transepithelial electrical resistance (TEER) values before and after the permeability assay in HBSS. Stable TEER values indicate that EV transport did not compromise epithelial barrier integrity (ns, *p* > 0.05). For experiments in the inflamed coculture, MSC-EVs were applied either to the apical or basolateral compartment (final concentration 1 × 10^9^ particles/mL). Both routes of EV administration significantly reduced the production of (f) TNF-α and (g) IL-6 in macrophages after 6 h (^1^**** and ^3^**** *p* < 0.0001) and 12 h (^2^**** and ^4^**** *p* < 0.0001). (h) Epithelial barrier integrity in the inflamed coculture measured by TEER. A characteristic feature of the inflamed model is a TEER reduction greater than 20% relative to baseline Caco-2 monoculture values (0 h). MSC-EVs applied to the apical and basolateral compartments of the coculture significantly increased TEER values after 16 h (^5^**** *p* < 0.0001). Data are presented as mean ± SD (*n* = 3). (c) One-way ANOVA with post-hoc Dunnett’s test. (e) One-way ANOVA with post-hoc Tukey’s test. (f), (g), and (h) Two-way ANOVA with post-hoc Dunnett’s test. (ns, *p* > 0.05; **** *p* < 0.0001).

To determine whether MSC-EVs could cross the intestinal epithelium, fluorescently labeled EVs were applied to the apical compartment, and transport across differentiated Caco-2 monolayers was quantified in the basolateral compartment over 3 h. The majority of EV transport occurred during the first 30 min, after which the transport rate gradually declined, likely due to reduced concentration gradients between the apical and basolateral compartments and EV uptake (Figure 3b). The mean particle size of transported EVs measured by NTA remained comparable to that of the applied EVs (Figure 3c), and cryogenic electron microscopy further confirmed the presence of structurally intact vesicles following transport across the intestinal epithelial monolayer (Figure 3d). TEER values remained stable before and after the permeability assay, indicating that EV transport did not compromise epithelial barrier integrity (Figure 3e).

Having established that MSC-EVs could cross differentiated Caco-2 monolayers while retaining structural integrity, we subsequently evaluated their anti-inflammatory activity in the inflamed coculture model. For the coculture experiments, differentiated Caco-2 cells were primed with a cytokine cocktail (TNF-α, IFN-γ and IL-1β) and J774A.1 macrophages were separately seeded and stimulated with LPS and IFN-γ. After 24 h, the two cell lines were combined into a coculture, with primed Caco-2 epithelial cells in the apical compartment and activated J774A.1 macrophages in the basolateral compartment (Figure 3a). This inflamed coculture was characterized by elevated TNF-α and IL-6 levels and loss of epithelial barrier integrity.

MSC-EVs (10^9^ particles/mL) were applied to the inflamed coculture either in the basolateral compartment, facing activated J774A.1 macrophages, or in the apical compartment, facing the primed Caco-2 monolayer (Figure 3a). Basolateral application represents intravenous administration, where EVs reach the subepithelial space (including immune cells) from the circulation, whereas apical application models oral administration, where EVs access the intestinal epithelium from the luminal side. Both routes of administration induced comparable effects in the inflamed coculture. After 6 h of incubation, MSC-EVs significantly reduced TNF-α levels 2-fold (Figure 3f) and IL-6 levels 3-fold (Figure 3g) in the basolateral compartment, and these effects persisted after 12 h. Importantly, EVs applied apically exerted anti-inflammatory activity equivalent to EVs applied directly to macrophages basolaterally, suggesting that EVs remained biologically functional following transport across the intestinal epithelial monolayer. MSC-EVs were also internalized by differentiated Caco-2 epithelial cells (Figure S5) and J774A.1 macrophages (Figure S6), supporting direct interactions of EVs with both epithelial and immune cell populations within the coculture model. After 12 h of EV treatment, TEER values began to increase in the Caco-2 epithelial monolayers and were restored to within 10% of healthy coculture values by 16 h (Figure 3h). Apical EV administration re-established epithelial barrier integrity to the same extent as basolateral administration.

These results demonstrate that apically administered MSC-EVs can cross intestinal epithelial monolayers while retaining structural integrity and anti-inflammatory activity, suggesting that oral delivery of MSC-EVs may represent a promising therapeutic approach for intestinal inflammation.

### MSC-EVs Are Not Stable in Gastrointestinal Fluids

Stability in the gastrointestinal tract is an essential prerequisite for oral administration. To investigate the stability of MSC-EVs for oral delivery, we first incubated the EVs with simulated gastric fluid (GF), followed by simulated intestinal fluid (IF) to mimic digestion in vitro.

EVs exposed to GF plus pepsin and IF plus pancreatin showed a significant decrease in particle size distribution, as indicated by mean size, mode, and percentiles D10, D50, and D90 (Figure 4a). MSC-EVs incubated with GF and IF also exhibited a significant reduction in particle concentration (4-fold) (Figure 4b) and in the percentage of EV subpopulations positive for TSG101 (2.5-fold) and CD81 (8-fold) (Figure 4c). Nanoflow cytometry scatter plots showed fewer detected events and a higher proportion of unstained nanoparticles in GF and IF (94.3%) than EVs in water (61.4%) (Figure 4d). Simulated gastrointestinal fluids had a disruptive effect on EVs comparable to sodium dodecyl sulfate (SDS), a surfactant that induces vesicle lysis. MSC-EVs were also visualized at different stages of incubation with GF and IF in the presence of digestive enzymes using cryogenic electron microscopy (Figure 4e). After 30 min, structural changes were evident and vesicles appeared nonspherical with disrupted or broken bilayer membranes (Figure 4ei,ii). After 1 h, vesicles had completely lost structural integrity and images revealed distorted membrane remnants (Figure 4eiii). Collectively, these results demonstrate that MSC-EVs are disintegrated in gastrointestinal fluids and are therefore not stable for oral administration without appropriate formulation.

**Figure 4:**
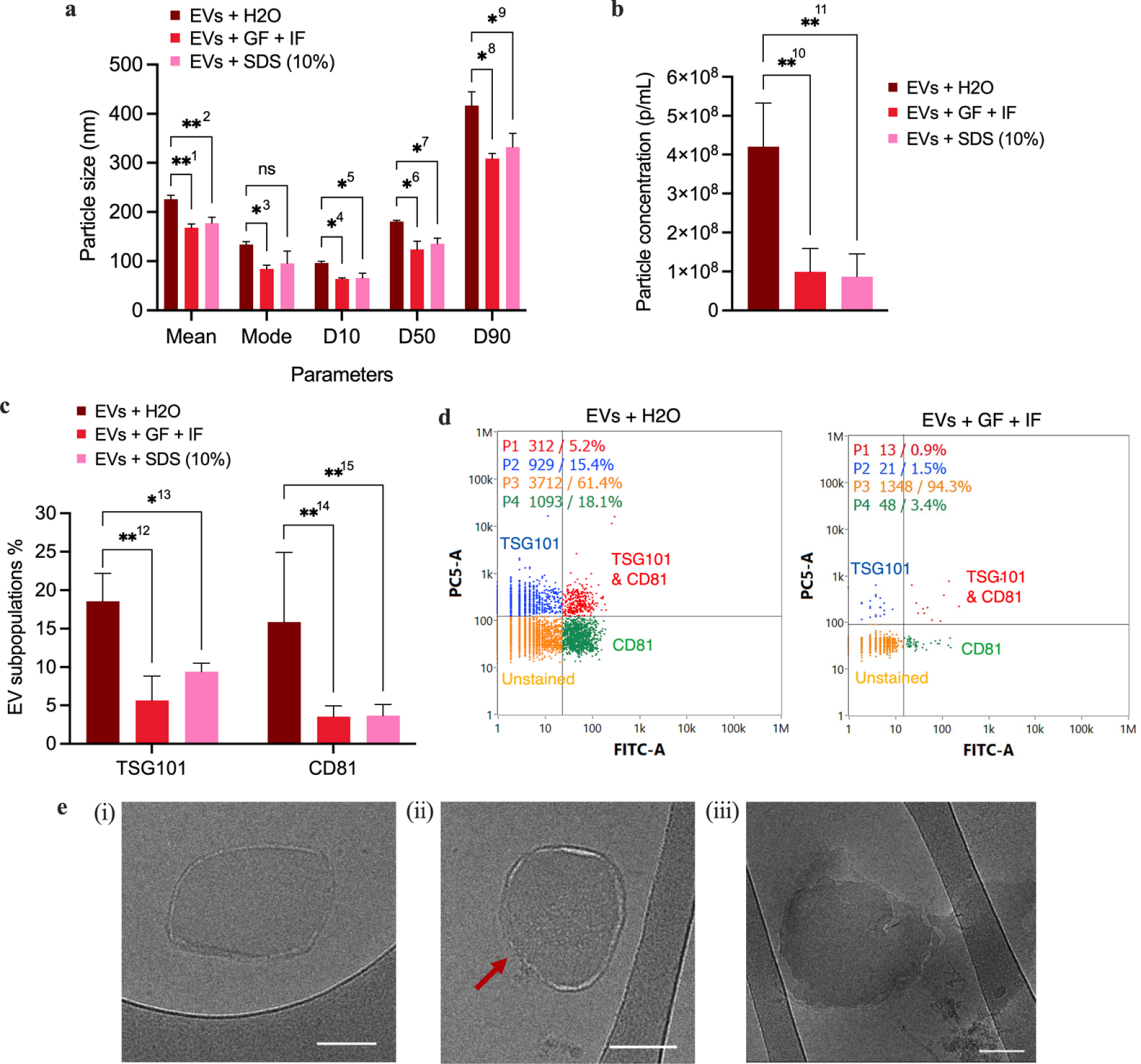
**Stability of MSC-EVs in gastrointestinal fluids.** MSC-EVs were incubated in simulated gastric fluid (GF) followed by simulated intestinal fluid (IF) to mimic digestion in vitro. (a) Particle size distribution of MSC-EVs measured by nanoparticle tracking analysis (NTA). All size parameters significantly decreased after incubation with GF and pepsin + IF and pancreatin: mean (^1^** *p* = 0.0020), mode (^3^* *p* = 0.0153), D10 (^4^* *p* = 0.0119), D50 (^6^* *p* = 0.0361), and D90 (^8^* *p* = 0.0227). 10% sodium dodecyl sulfate (SDS) was used as a positive control for EV disintegration and showed a significant reduction in mean (^2^** *p* = 0.0089), D10 (^5^* *p* = 0.0431), D50 (^7^* *p* = 0.0249) and D90 (^9^* *p* = 0.0454). (b) Particle concentration of MSC-EVs measured by nanoflow cytometry. Incubation with GF and IF significantly reduced the particle concentration of EVs (^10^** *p* = 0.0052) to the same extent as 10% SDS (^11^** *p* = 0.0043). (c) Percentage of EV subpopulations positive for TSG101 and CD81 relative to total particles detected by nanoflow cytometry. Incubation of EVs with GF and IF significantly reduced the percentage of EVs expressing the cytosolic protein TSG101 (^12^** *p* = 0.0057) and the transmembrane protein CD81 (^14^** *p* = 0.0078). 10% SDS also significantly reduced TSG101-positive EVs (^13^* *p* = 0.0407) and CD81-positive EVs (^15^** *p* = 0.0084). (d) Representative dot plots showing gated populations of stained EVs based on fluorescence intensities, FITC-A: anti-CD81 (488) vs PC5-A: anti-TSG101 (647), measured with a flow nanoanalyzer (NanoFCM). P1: double-positive for TSG101 and CD81; P2: TSG101-only positive; P3: double-negative (unstained); and P4: CD81-only positive. The subpopulation of EVs positive for TSG101 and/or CD81 decreased following incubation with GF and IF. (e) Cryogenic electron microscopy (cryo-EM) images of MSC-EVs at different stages of incubation with GF and IF and digestive enzymes pepsin and pancreatin. Scale bar: 100 nm. After 30 min, (i) the vesicle morphology is no longer spherical and (ii) the bilayer membrane is ruptured (red arrow). (iii) After 1 h, the vesicle has completely lost its structural integrity, leaving behind irregular membrane debris. Data are presented as mean ± SD (*n* = 3). (a) and (c) Two-way ANOVA with post-hoc Dunnett’s test. (b) One-way ANOVA with post-hoc Dunnett’s test. (ns, *p* > 0.05; * *p* < 0.05; ** *p* < 0.01).

### Double-Coating Formulation Improves Gastrointestinal Stability and Enables Colon-Targeted Release of MSC-EVs

To facilitate oral delivery of MSC-EVs, we developed a double-coating formulation to protect the vesicles from degradation in the gastrointestinal (GI) environment and to target delivery to the colon. MSC-EVs were sequentially coated with chitosan as the first layer and Eudragit S-100 as the second layer to form chitosan-Eudragit-coated EVs, referred to as coated EVs (Figure 5a). Chitosan coating of EVs is driven by electrostatic attraction between the positively charged chitosan and the negatively charged EV surface. Following coating, the total number of particles in the solution was preserved, and the chitosan-coated EVs exhibited a positive surface charge and an increase in average particle size by 1.5–2-fold (Figure 5b). The structural differences between uncoated and chitosan-coated EVs were also visualized by cryogenic electron microscopy (Figure 5ci,ii, respectively). When anionic Eudragit S-100 is deposited onto chitosan-coated EVs, electrostatic attraction between the oppositely charged polymers leads to the formation of a polyelectrolyte complex on the EV surface. This interaction results in the assembly of a solid double-layer coating around the vesicles, causing the coated EVs to precipitate from the solution. Scanning electron microscopy (SEM) images revealed coated particles embedded within a surrounding polymeric matrix (Figures 5ciii and S9a).

**Figure 5:**
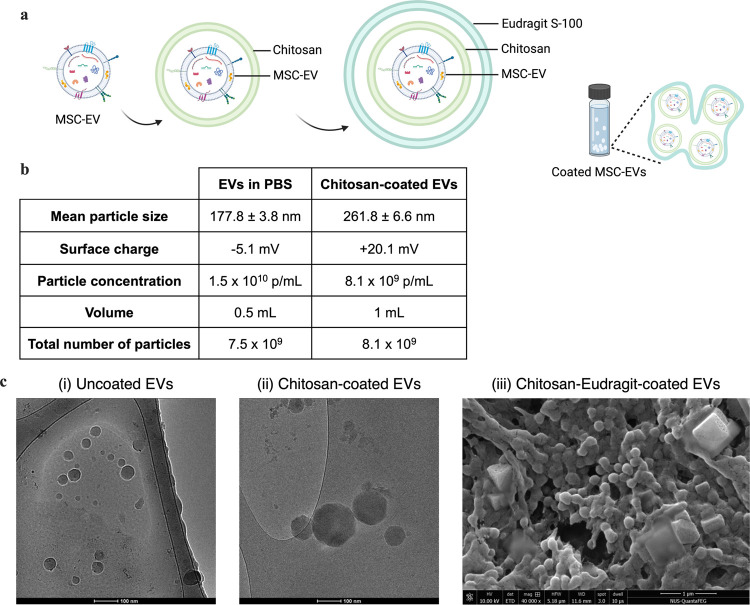
**Double-coating formulation of MSC-EVs.** (a) Schematic illustration of the coating process. MSC-EVs were sequentially coated with chitosan (first layer) and Eudragit S-100 (second layer) to form chitosan-Eudragit-coated EVs, referred to as coated EVs. Cationic chitosan adheres to negatively charged EVs, then anionic Eudragit binds to the chitosan layer, thereby forming a sequential coating structure through electrostatic attraction. Created using BioRender.com. (b) Comparison between uncoated EVs and chitosan-coated EVs. Particle size and concentration were analyzed using nanoparticle tracking analysis (NTA), and surface charge was measured by zeta potential. (c) Microscopy images showing each step of the coating process. (i) Uncoated EVs and (ii) chitosan-coated EVs were visualized by cryogenic electron microscopy (cryo-EM). Scale bar: 100 nm. (iii) Chitosan-Eudragit-coated EVs (referred to as coated EVs) were imaged with scanning electron microscopy (SEM). Scale bar: 1 μm. The SEM image shows coated particles embedded within a surrounding matrix structure.

The double-coating formulation employs a sequential pH- and bacteria-responsive system to enable targeted delivery to the colon. Chitosan was selected as the inner layer because it is specifically degraded by microbial enzymes produced by colonic bacteria. Its mucoadhesive properties^45^ may also enhance retention and release of EVs on mucosal surfaces of the colon. As a cationic polymer, chitosan stabilizes the coating by acting as an intermediate electrostatic layer between the negatively charged EV surface and the anionic Eudragit, promoting the assembly of a stable double-layer structure. Eudragit S-100 was selected as the outer layer due to its resistance to pH conditions in the stomach and proximal small intestine. The methacrylate copolymer is widely used in enteric coatings^46^ as it only dissolves at pH above 7.0, enabling drug release in the ileocolonic region,^47^ which can increase the local bioavailability of EVs in the colon. The stability of coated EVs in the upper GI tract and colon-targeted release of EVs are illustrated in Figure 6a.

**Figure 6:**
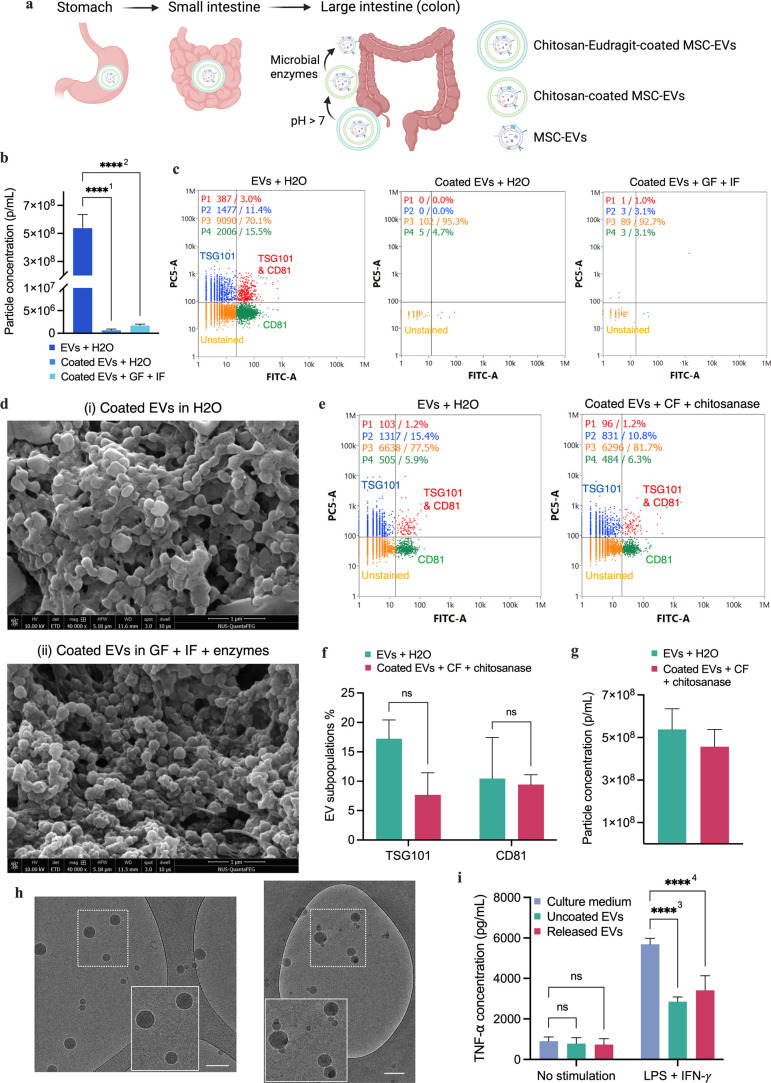
**Stability of coated EVs in gastrointestinal environment and colon-targeted EV release.** (a) Schematic illustration of the stability of coated EVs in the gastrointestinal (GI) tract and sequential release in the colon. Coated MSC-EVs remain intact in the stomach and small intestine. Upon reaching the ileocolonic region, the outer Eudragit layer dissolves at pH > 7 and the inner chitosan layer is subsequently degraded by microbial enzymes in the colon, releasing EVs at the target site. Created using BioRender.com. (b) Particle concentration in the supernatant of coated EVs. Coated EVs both in water (pH 6.5) and after incubation with simulated gastric fluid (GF) and intestinal fluid (IF) showed significantly lower particle numbers in solution compared with uncoated EVs (^1^**** and ^2^**** *p* < 0.0001). The particle concentration for coated EVs was below the detection limit (1 × 10^8^ p/mL) for the flow nanoanalyzer (NanoFCM). (c) Representative NanoFCM dot plots showing the percentage of EVs stained for TSG101 and/or CD81. In the supernatant of coated EVs both in water (pH 6.5) and GF + IF, only a small number of events (<120) were recorded and ≥90% of the particles detected were unstained. (d) Scanning electron microscopy (SEM) images of coated EVs in (i) water (pH 6.5) and (ii) GF + IF with the digestive enzymes pepsin and pancreatin, showing preservation of the coating morphology. (e) Representative dot plots showing gated populations in stained uncoated EVs and coated EVs incubated with simulated colonic fluid (CF) and chitosanase based on fluorescence intensities, FITC-A: anti-CD81 (488) vs PC5-A: anti-TSG101 (647), measured with a flow nanoanalyzer (NanoFCM). Similar dot plot distributions were observed for uncoated EVs and EVs released from the coating formulation. P1: double-positive for TSG101 and CD81; P2: TSG101-only positive; P3: double-negative (unstained); and P4: CD81-only positive. (f) Percentage of EV subpopulations positive for TSG101 and CD81 relative to total particles detected by nanoflow cytometry. The proportion of TSG101- and CD81-positive EVs released from coated EVs in CF + chitosanase were not significantly different from uncoated EVs (ns, *p* > 0.05). (g) Particle concentration measured by nanoflow cytometry. 85% of particles were released from the coating within a 6 h incubation with CF + chitosanase. (h) Cryogenic electron microscopy (cryo-EM) images of vesicles released from the coating formulation showing preservation of spherical morphology. Scale bar: 100 nm. (i) Effect of released EVs on TNF-α levels in J774A.1 macrophages measured by ELISA. EVs released from the coating formulation did not activate unstimulated macrophages and reduced TNF-α production in LPS/IFN-γ-activated macrophages to the same extent as uncoated EVs (^3^**** and ^4^**** *p* < 0.0001). Data are presented as mean ± SD (*n* = 3). (b) One-way ANOVA with post-hoc Dunnett’s test. (f) Multiple paired *t* tests. (i) Two-way ANOVA with post-hoc Dunnett’s test. (ns, *p* > 0.05; **** *p* < 0.0001).

To assess stability in the upper GI tract, coated EVs were incubated with simulated gastric fluid (GF) followed by simulated intestinal fluid (IF). The supernatant was then analyzed to determine whether EVs had been released from the coating. The particle concentration in GF and IF was below the detection limit for the NanoFCM flow nanoanalyzer (1 × 10^8^ particles/mL) and similar to background noise, indicating that the coating did not degrade and remained stable around the EVs (Figure 6b). Consistently, nanoflow cytometry scatter plots showed that of the few events (<120) detected, ≥90% of particles were negative for TSG101 and/or CD81 (Figure 6c), suggesting that they may have been coating debris rather than EVs. SEM images obtained before and after incubation with GF and IF in the presence of digestive enzymes pepsin and pancreatin further confirmed that the coating remained intact and protected EVs from degradation, as individual particles were still visible within the coating matrix (Figures 6d and S9b).

To evaluate release of EVs in the colon, coated EVs were incubated with simulated colonic fluid (CF) and chitosanase to mimic enzymatic degradation of chitosan. Analysis by nanoflow cytometry revealed the presence of TSG101- and CD81-positive EVs (Figure 6e) at percentages comparable to uncoated EVs (Figure 6f). Particle concentration measurements showed that ∼85% of EVs were released from the coating after 6 h (Figure 6g) and cryo-EM images confirmed that the released EVs preserved their structural integrity (Figure 6h). EVs released from the coating formulation also retained their biological activity as evidenced by the reduction in TNF-α levels in activated J774A.1 macrophages (Figure 6i).

These results demonstrate that the double-coating formulation remains intact under conditions simulating the stomach and small intestine, preventing premature release or degradation of EVs, while enabling rapid EV release in conditions mimicking the colon.

### Orally Administered Coated MSC-EVs Target the Colon and Alleviate Disease Severity in a DSS-Induced Colitis Mouse Model

We used a dextran sodium sulfate (DSS)-induced colitis mouse model to determine the biodistribution of MSC-EVs in vivo. DSS administration in mice induces mucosal inflammation localized to the colon, mimicking key features of human ulcerative colitis.^48–50^ The double-coating formulation increased EV accumulation in the colon 24 h after oral administration compared with uncoated EVs (Figure 7a,b), indicating enhanced gastrointestinal stability and colon-targeted delivery. Coated EVs also showed lower liver localization than uncoated EVs following oral administration (Figure S10), suggesting reduced systemic absorption and hepatic accumulation. Oral delivery of coated EVs was associated with higher colonic and lower hepatic signal intensities relative to EVs administered intravenously.

**Figure 7:**
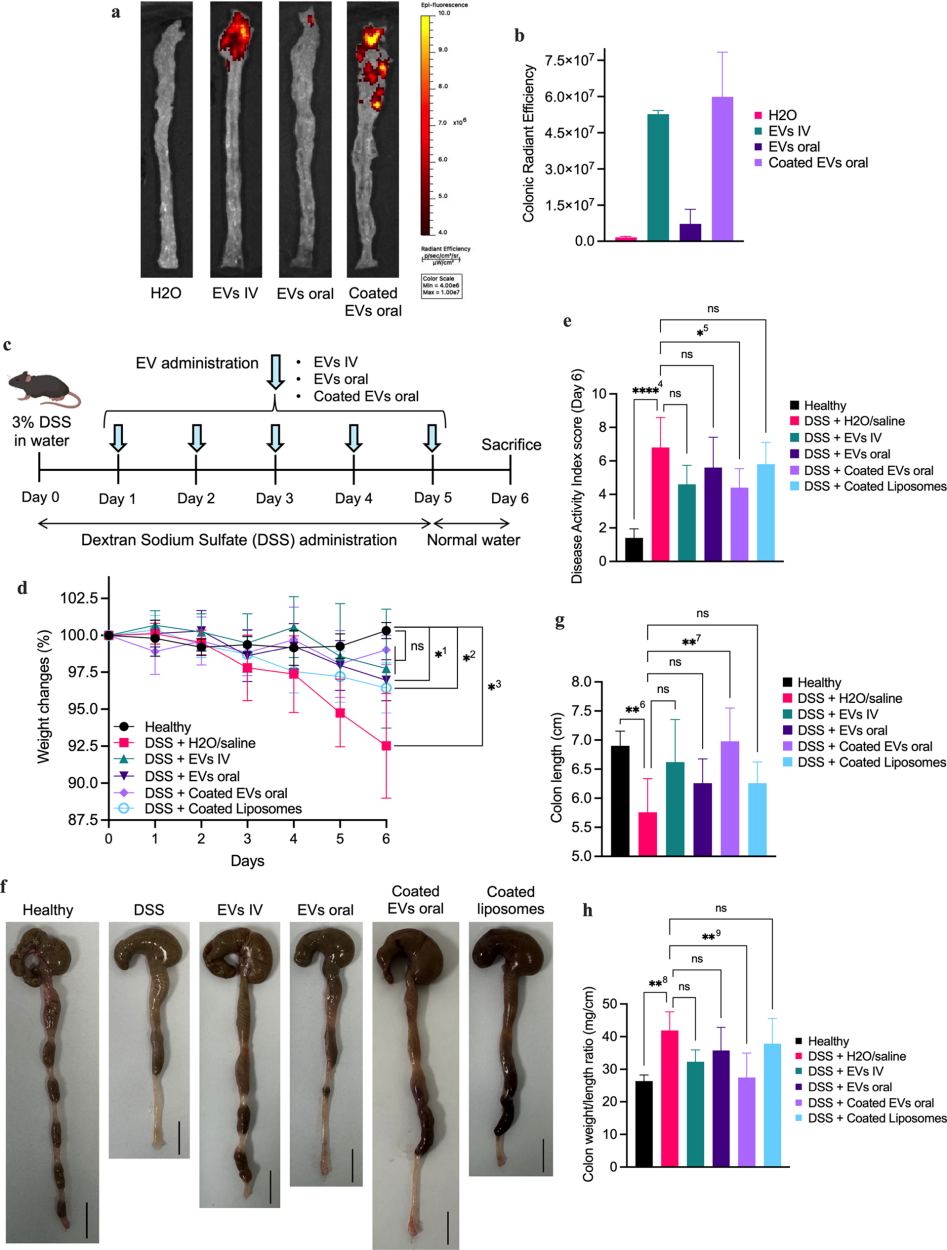
**Biodistribution and therapeutic efficacy of coated MSC-EVs in a DSS-induced colitis mouse model.** (a) Biodistribution of MSC-EVs in the colon of dextran sodium sulfate (DSS)-treated mice 24 h after EV administration. MSC-EVs labeled with wheat germ agglutinin-Alexa Fluor 680 (WGA-AF680) were administered via IV injection and oral gavage for uncoated and coated EVs, and the fluorescence in excised colons was imaged using an in vivo imaging system (IVIS, Revvity). (b) Quantification of EV fluorescence signal (radiant efficiency) in the colon 24 h after administration. Coating of EVs increased colonic fluorescence intensity compared with uncoated EVs administered orally or intravenously. Data are presented as mean ± SD from two independent experiments (*n* = 2). (c) Study design for DSS-induced colitis in mice and the dosing schedule of MSC-EVs. 3% (w/v) DSS was administered in drinking water for 5 days to induce acute colitis in mice. MSC-EVs (1 × 10^9^ particles/mouse/day) were given on days 1–5 via intravenous (IV) injection (EVs IV) and by oral gavage for uncoated EVs (EVs oral) and coated EVs (coated EVs oral). Mice were sacrificed on day 6. (d) Weight changes in mice over 6 days. Mice treated with EVs via IV injection and coated EVs administered orally showed minimal changes in body weight, similar to healthy mice. There was significant body weight loss in mice treated with uncoated EVs orally (^1^* *p* = 0.0122), coated liposomes (^2^* *p* = 0.0164), and in DSS control mice (^3^* *p* = 0.0231). (e) Disease activity index (DAI) scores on day 6. DAI combines scores for weight loss, stool consistency, and fecal occult blood. The DAI score was the highest for DSS control mice and the lowest for healthy mice (^4^**** *p* < 0.0001). Mice treated with coated EVs orally were the only treatment group that showed a significantly lower DAI score (^5^* *p* = 0.0407). (EVs IV *p* = 0.0670). (f) Photographs of colons on day 6. Scale bar 1 cm. (g) Colon length measurements. Treatment with coated EVs significantly increased the colon length of DSS mice (^7^** *p* = 0.0041) and showed similar results as healthy colons (^6^** *p* = 0.0075). (EVs IV *p* = 0.0539). (h) Colon weight to length ratio. Mice treated with coated EVs showed a significant reduction in the colon weight/length ratio (^9^** *p* = 0.0038) to the same extent as healthy colons (^8^** *p* = 0.0019). (EVs IV *p* = 0.0705). Data are presented as mean ± SD (*n* = 5). (d) Two-way ANOVA with Dunnett’s test. (e), (g), and (h) One-way ANOVA with Dunnett’s test. (ns, *p* > 0.05; * *p* < 0.05; ** *p* < 0.01; **** *p* < 0.0001).

We conducted preliminary in vivo studies to evaluate the therapeutic efficacy of coated MSC-EVs. Acute colitis was induced in male C57BL/6J mice by adding 3% DSS to drinking water for 5 days. MSC-EVs (1 × 10^9^ particles/mouse) were administered on days 1–5 via intravenous (IV) injection and by oral gavage (uncoated EVs and coated EVs) (Figure 7c). Mice were monitored daily and clinical signs, including weight loss, diarrhea and presence of blood in stools, were scored and summed to generate the disease activity index (DAI). On day 6, mice were euthanized and colons were collected for analysis.

DSS treatment resulted in significant body weight loss after 6 days, an effect that was also observed in mice given uncoated MSC-EVs orally (Figure 7d). By contrast, mice that received coated EVs orally and EVs via IV injection maintained their body weight throughout the study, similar to healthy controls. However, only coated EVs significantly reduced DAI scores on day 6 compared with other EV treatment groups, indicating improved clinical outcomes and reduced colitis severity (Figure 7e). At necropsy, the colons of each group were photographed (Figure 7f). DSS exposure shortens and thickens the colon as a result of inflammation and damage to the intestinal mucosa. Treatment with coated EVs significantly increased colon length (Figure 7g) and reduced colon weight/length ratio (Figure 7h), normalizing these parameters to values observed in healthy controls. By comparison, uncoated EVs administered orally produced outcomes similar to the DSS control group, emphasizing the importance of formulation in preserving the biological activity of MSC-EVs. Additionally, coated EVs induced a stronger therapeutic response than intravenous EVs administered at the same relatively low dose, suggesting that oral delivery may offer advantages when the site of action is localized to the colon. The weaker therapeutic effect observed with intravenous EVs may reflect rapid systemic clearance and limited targeting or retention at the site of inflammation. The enhanced efficacy of coated EVs may also reflect a synergistic contribution from chitosan, which has intrinsic anti-inflammatory properties.^51^ To confirm that the therapeutic effect of coated EVs was predominantly mediated by EVs rather than the coating, we included an additional group treated with coated liposomes. These mice exhibited responses similar to the DSS control group, including weight loss, elevated DAI scores, and colon shortening and thickening, demonstrating that the coating alone did not confer therapeutic benefit in DSS-induced colitis.

Overall, our study explores the oral delivery of MSC-EVs as a potential therapeutic strategy for intestinal inflammation. We conducted a systematic evaluation of the gastrointestinal stability of MSC-EVs and demonstrated that they disintegrate in the gastrointestinal environment, resulting in a loss of biological activity. To facilitate oral delivery, we developed a chitosan-Eudragit double-coating formulation that improves EV stability in GI fluids and targets delivery to the colon. This coating system protected MSC-EVs from degradation under digestive conditions and enabled the release of structurally intact, biologically active vesicles in simulated colonic fluid. MSC-EVs were also shown to cross intestinal epithelial monolayers while retaining structural integrity and anti-inflammatory activity in vitro. In vivo, orally administered coated EVs elicited a therapeutic response in the colitis model that was not observed with uncoated EVs, suggesting successful release of functional vesicles within the inflamed colon. Our preliminary in vivo studies further indicated that orally administered coated EVs exerted a stronger therapeutic effect than intravenously delivered EVs. Additional biodistribution and pharmacokinetic studies would be needed to determine whether this enhanced efficacy is due to improved targeting, greater retention, or both, within inflamed intestinal tissues.

Although MSC-EVs have been extensively studied in colitis models of IBD via systemic administration, oral delivery remains underexplored. Prior to this work, only two studies (Gan et al.^52^ and Deng et al.^53^) reported the therapeutic efficacy of orally administered, formulated MSC-EVs in colitis. Our findings therefore contribute to the emerging evidence supporting oral delivery of MSC-EVs as a novel therapeutic approach in IBD.

Our in vivo evaluation was restricted to macroscopic end points, including disease activity index and colon morphology, which offer only a partial view of therapeutic efficacy. More comprehensive characterization, such as histological assessment and cytokine profiling, is required to substantiate these findings and determine the extent of mucosal healing and inflammation resolution.

## Conclusions

MSC-EVs possess strong therapeutic potential for IBD owing to their regenerative and immunomodulatory properties. Effective treatment of IBD requires localized drug delivery to the intestine, making oral administration an attractive and patient-friendly route. In this study, we demonstrated that MSC-EVs are unstable in gastrointestinal fluids, underscoring the need for an optimized delivery system to preserve vesicle integrity and function during oral administration. We developed a double-coating formulation that protects MSC-EVs from degradation in the GI environment and targets delivery to the colon. Our in vivo findings suggest that, with suitable formulation, oral delivery of MSC-EVs may offer a promising strategy for treating intestinal inflammation.

## Experimental Section

### Cell Culture

Human bone marrow mesenchymal stem cells (hBM-MSCs) were purchased from Lonza (product #PT-2501) in 2022 and 2024, certified mycoplasma-free, and used between passages 3–5. The primary cells were cultured in Dulbecco’s modified eagle medium (DMEM) containing 1 g/L glucose (product #31885–023, Thermo Fisher Scientific) supplemented with 10% (v/v) EV-depleted fetal bovine serum (FBS; product #F9665, Sigma-Aldrich) and 1% (v/v) antibiotic-antimycotic solution 100X containing penicillin, streptomycin and amphotericin B (product #15240–062, Thermo Fisher Scientific). EV-depleted FBS was prepared using the gold standard protocol^54^ by subjecting FBS to ultracentrifugation (Optima XPN, Beckman Coulter) at 100,000 *g* in a fixed-angle rotor (45 Ti, Beckman Coulter) for 18 h at 4 °C. The FBS supernatant was then collected and sterile filtered using 0.22 μm filters before use in cell culture medium.

Human epithelial colorectal adenocarcinoma (Caco-2; RRID:CVCL_0025) and murine J774A.1 macrophage (RRID:CVCL_0358) were purchased from the European Collection of Authenticated Cell Cultures in 2020 and 2021, respectively, and certified mycoplasma-free. Caco-2 cells were used between passages 25–50 and J774A.1 cells were used between passages 10–30. Both cell lines were cultured in DMEM containing 4.5 g/L glucose (product #41966–029, Thermo Fisher Scientific) supplemented with 10% (v/v) FBS, 1% (v/v) nonessential amino acids (NEAA; product #25–025-Cl, Corning) and 1% (v/v) antibiotic-antimycotic solution 100X containing penicillin, streptomycin and amphotericin B.

All cells were maintained at 37 °C in a humidified incubator containing 5% CO_2_.

### Isolation of MSC-EVs

EVs were isolated using a one-step sucrose cushion ultracentrifugation method, as described previously^55^ (Figure S1). The conditioned medium (CM) of hBM-MSCs was harvested and precleared of dead cells and cellular debris through several rounds of differential centrifugation: 500 *g* for 5 min at 4 °C twice, then 2000 *g* for 15 min at 4 °C; followed by filtration of the supernatant through 0.22 μm sterile filter. Precleared CM was either stored at −80 °C or directly processed for EV isolation using the Optima XPN ultracentrifuge (Beckman Coulter). Thickwall polycarbonate ultracentrifuge tubes (product #355631, Beckman Coulter) were filled with 22.5 mL of precleared CM, then 3 mL of 25% (w/w) sucrose solution (product #S/8600/60, Fisher Scientific) prepared in deuterium oxide (D_2_O; product #151882, Sigma-Aldrich) was slowly layered below the conditioned medium using a glass Pasteur pipet. The ultracentrifuge tubes were placed in a swing-out rotor (SW32 Ti or SW28 Ti, Beckman Coulter) and subjected to ultracentrifugation at 100,000 *g* for 1.5 h at 4 °C. The sucrose cushion layer containing EVs was then collected (2 mL per tube) and added to filtered PBS in polycarbonate ultracentrifuge tubes (product #355622 or #355618, Beckman Coulter). The ultracentrifuge tubes were subjected to ultracentrifugation in a fixed-angle rotor (45 Ti or 70 Ti, Beckman Coulter) at 100,000 *g* for 1.5 h at 4 °C as a washing step for EV purification. The supernatant was then discarded and the EV pellet was resuspended in filtered PBS before storage at 4 °C for short-term use or −80 °C for long-term use.

### Characterization of MSC-EVs

#### Nanoparticle Tracking Analysis (NTA)

The size distribution and concentration of EVs were measured by NTA using a NanoSight instrument (LM10 or NS300, Malvern Panalytical). EV samples were diluted in filtered PBS to obtain 20–80 particles in the viewing frame for optimal tracking. The samples were injected into the laser viewing module and the camera level was adjusted to 14–15, with screen gain 1. The analysis detection threshold was set at 5–7. For each sample, video recordings of 30–60 s duration were captured and analyzed using the NanoSight NTA software. A size distribution histogram was generated based on the Brownian motion of the particles in each sample.

#### Bicinchoninic Acid (BCA) Assay

The total protein concentration of EVs was quantified using the QuantiPro BCA assay kit (product #QPBCA-1KT, Sigma-Aldrich). EV samples and serially diluted bovine serum albumin (BSA) standards (0–20 μg/mL) prepared in filtered PBS were added to a 96-well plate (50 μL/well). The working reagent (prepared according to the manufacturer’s instructions) was then added to the wells in equal volume (50 μL/well) and the plate was incubated at 60 °C for 1 h. The absorbance was measured at 562 nm using an Infinite 200 Pro plate reader (Tecan) and a BSA standard curve was used to determine the protein concentration of EV samples.

#### Zeta Potential

The zeta potential of EVs was measured at 25 °C using a Zetasizer instrument (Zetasizer Nano or Ultra, Malvern Panalytical). EV samples were diluted in filtered PBS (minimum concentration 1 × 10^11^ particles/ml) prior to loading the samples in folded capillary zeta cells (product #DTS1070, Malvern Panalytical) and analyzed using the ZS Explorer software.

#### Cryogenic Electron Microscopy (cryo-EM)

A volume of 3.5 μL of EV samples (minimum concentration 1 × 10^9^ particles/mL) was applied twice to a Tedpella lacey Formvar/carbon copper grid (Ted Pella, Inc.) that was glow-discharged in air for 60 s. Then the grid was blotted for 1.5 s (blot force 1) at 22 °C and 100% humidity, followed by a plunge into liquid ethane using FEI Vitrobot Mark IV. The micrographs were recorded using a 200 kV Tecnai Arctica cryo-transmission electron microscope equipped with a Falcon 3EC direct electron detector. Images were collected at a magnification of 39,000× and 53,000×, yielding the pixel sizes of 2.79 and 2.01 Ångstroms per pixel (Å/px), respectively.

#### Exosome Antibody Array

The presence of EV-associated proteins was detected using the Exo-Check Exosome Antibody Array kit (product #EXORAY200A-4, System BioSciences) following the manufacturer’s protocol. The array had eight positive markers: tetraspanins CD63 and CD81, epithelial cell adhesion molecule (EpCAM), annexin A5 (ANXA5), tumor susceptibility gene 101 (TSG101), flotillin-1 (FLOT1), intercellular adhesion molecule 1 (ICAM), programmed cell death 6 interacting protein (ALIX). The array also contained four controls: cis-Golgi matrix protein (GM130) as a negative marker for cellular contamination during EV isolation, two positive controls and a blank control.

#### Super-Resolution Microscopy

The EV Profiler 2 kit (Oxford Nanoimaging) was used for visualizing and phenotyping single EVs. EVs were stained with a tetraspanin antibody panel consisting of anti-CD63 (561), anti-CD81 (647) and anti-CD9 (488). Samples were processed according to manufacturer’s instructions to immobilize the stained EVs on the assay chips. Three fields of view were recorded for each sample and super-resolution images were acquired using direct stochastic optical reconstruction microscopy (dSTORM) on a Nanoimager instrument (Oxford Nanoimaging). EV clusters were filtered based on circularity, density of localizations and area to remove background and non-EV-related clusters. Colocalizations of the different tetraspanins were analyzed using the CODI platform (Oxford Nanoimaging).

### Cell Proliferation Assay

The MTS assay was used to assess cell viability, metabolic activity and proliferation. Caco-2 cells were seeded at different densities (5000–150,000 cells/well) in 48-well plates (Thermo Fisher Scientific) and incubated overnight (12 h) at 37 °C to allow cells to adhere to the wells. The next day, the cells were washed with PBS before 0.2 mL of serum-free medium and 20 μL of MTS reagent (CellTiter 96 AQ_ueous_ One Solution, product #G3581, Promega) were added to the wells. Cells with active mitochondrial metabolism convert the MTS tetrazolium reagent into a purple-colored formazan product that is soluble in cell culture medium and can be quantified by measuring absorbance at 490 nm.^56^ After 2 h incubation at 37 °C, the absorbance of the formazan product generated in the wells was measured at 490 nm using an Infinite 200 Pro plate reader (Tecan). The absorbance of formazan was directly proportional to the number of viable Caco-2 cells and a standard curve was constructed to plot the absorbance at 490 nm against different Caco-2 cell densities per well (Figure S2).

For the cell proliferation assay, Caco-2 cells were seeded in 48-well plates (Thermo Fisher Scientific) at a density of 1.5 × 10^5^ cells/mL (0.2 mL/well) and incubated at 37 °C for 24 h. The next day, culture medium was removed from the wells and Caco-2 cells were incubated with increasing concentrations (10^7^, 10^9^, and 10^11^ particles/mL) of MSC-EVs prepared in serum-free culture medium (0.2 mL/well). Serum-free cell culture medium was used as a control and 10% (v/v) dimethyl sulfoxide (DMSO; product #D2438, Sigma-Aldrich), also in serum-free culture medium, was used as a negative control for cell proliferation since it is toxic to cells. The MTS assay was performed daily for 4 consecutive days. The sample and control solutions were discarded from the wells and the cells were washed with PBS, then 0.2 mL of serum-free medium was added to each well followed by 20 μL of MTS reagent. The plate was incubated for 2 h at 37 °C and the absorbance at 490 nm was measured using an Infinite 200 Pro plate reader (Tecan). Caco-2 cell density/well was determined using the constructed standard curve.

### Wound Healing Assay

Culture-inserts (product #81176, Ibidi) consisting of 2 wells separated by a wall were placed in 12-well plates (Thermo Fisher Scientific). Caco-2 cells were seeded in each well of the culture-insert at a density of 7 × 10^5^ cells/mL (70 μL/well) and incubated at 37 °C for 24 h. The following day, culture-inserts were removed using sterile tweezers, creating a cell-free 500 μm gap between the two cell layers, and the wells were washed with PBS to remove cell debris. Caco-2 cells were then incubated with 1 mL of serum-free culture medium containing increasing concentrations (10^7^, 10^9^, and 10^11^ particles/mL) of MSC-EVs. Serum-free cell culture medium was used as a control. Images of the wounds were captured at regular intervals (0, 24, 48, and 72 h) using an Eclipse Ts2R inverted microscope (Nikon) on a 10× objective. The images were then processed using a *wound_healing_size_tool* plugin^57^ for ImageJ to measure the wound area and average wound width. Wound healing was quantified using two metrics: percentage of area reduction or wound closure (eq 1) and rate of cell migration (*R*_M_; eq 2).^40^

$$\mathrm{Wound}\;\mathrm{closure}\%=\left(\frac{A_{t=0}-A_{t=\Delta t}}{A_{t=0}}\right)\times 100\%$$ 1

Where *A*_*t*=0_ is the initial wound area and *A*_*t*=Δ*t*_ is the wound area after *n* h, both in μm^2^.

$$R_{M}=\frac{W_{i}-W_{f}}{t}$$ 2

*R*_M_ is the rate of cell migration (μm/h). *W*_i_ is the initial wound width, *W*_f_ is the final wound width, both in μm, and *t* is the duration of the migration in h.

### Pro-Inflammatory Cytokine Assay

J774A.1 macrophages were seeded in 48-well plates (Thermo Fisher Scientific) at a density of 1 × 10^5^ cells/mL (0.4 mL/well) in culture medium containing 100 ng/mL lipopolysaccharide (LPS) from *Escherichia coli* O111:B4 (product #L2630, Sigma-Aldrich) and 25 ng/mL interferon-γ (IFN-γ; product #GFH77, Cell Guidance Systems) to induce inflammation. The cells were incubated at 37 °C for 24 h. The next day, activated macrophages were treated with increasing concentrations of MSC-EVs (final concentration in wells: 10^7^, 10^9^, and 10^11^ particles/mL) and incubated for a further 24 h. One μg/mL Dexamethasone (product #D4902, Sigma-Aldrich) was used as a positive control for anti-inflammatory activity and cell culture medium was used as a negative control. Cytokines (TNF-α and IL-6) released from J774A.1 macrophages were quantified using a Mouse Luminex Discovery assay (product #LXSAMSM, Bio-Techne), following the manufacturer’s protocol. The median fluorescence intensity was measured using a Luminex Flexmap3D Reader and standard curves for each cytokine were generated to quantify cytokine levels in the samples.

### Oxidative Stress Assay

J774A.1 macrophages were seeded in 24-well plates (Thermo Fisher Scientific) at a density of 2 × 10^5^ cells/mL in culture medium without antibiotics (0.5 mL/well) containing 100 ng/mL LPS (product #L2630, Sigma-Aldrich) and 100 nM phorbol 12-myristate 13-acetate (PMA; product #P8139, Sigma-Aldrich) to induce oxidative stress. The cells were incubated at 37 °C for 24 h. The next day, activated macrophages were treated with increasing concentrations of MSC-EVs (final concentration in wells: 10^7^, 10^9^, and 10^11^ particles/mL) and incubated for a further 24 h. Cellular oxidative stress was quantified by measuring total reactive oxygen species (ROS) using 2’,7’-dichlorodihydrofluorescein diacetate (DCFH-DA; product #D6883, Sigma-Aldrich). DCFH-DA is taken up by cells where cellular esterase cleaves off the acetyl groups, resulting in DCFH, which is then oxidized by ROS and converted to DCF, which emits green fluorescence that can be measured.^58^ J774A.1 macrophages were washed with DMEM, then stained with 0.5 mL DCFH-DA solution (50 μM) and incubated at 37 °C for 30 min. The cells were subsequently washed with DMEM and incubated with 0.2 mL RIPA buffer (product #R0278, Sigma-Aldrich) on ice for 5 min. The lysate was collected and centrifuged at 16000 *g* for 10 min at 4 °C. The supernatant was collected and the fluorescence (Ex/Em 485/530) was measured using an Infinite 200 Pro plate reader (Tecan). One mmol/L Nω-Nitro-l-arginine methyl ester (L-NAME; product #N5751, Sigma-Aldrich) was used as a positive control for antioxidant activity as it indirectly reduces cellular ROS production by preventing peroxynitrite formation through the inhibition of the enzyme nitric oxide synthase, which produces nitric oxide (NO). Cell culture medium without antibiotics was used as a negative control.

### Transport and Uptake of MSC-EVs In Vitro

#### Fluorescent Labeling of MSC-EVs

MSC-EVs were labeled with fluorescent wheat germ agglutinin (WGA) conjugates. Briefly, MSC-EVs were incubated with 0.1 mg/mL WGA-Alexa Fluor 488 (WGA-AF488; product #W11261, Thermo Fisher Scientific) or WGA-Alexa Fluor 680 (WGA-AF680; product #W32465, Thermo Fisher Scientific) at 37 °C for 30 min under gentle agitation, protected from light (Figure S3a). The labeled EVs were purified using Exo-spin columns (Exo-spin mini, product #EX03–50, Cell Guidance Systems) and the recovery of EVs was determined using nanoparticle tracking analysis (NTA) (Figure S3b). Prior to each assay, labeled EVs were analyzed by NTA to measure particle size and concentration for accurate in vitro dosing. For permeability studies, a fluorescence standard curve was also constructed to determine the particle concentration in samples.

#### Transport of MSC-EVs across Differentiated Caco-2 Epithelial Monolayers

The intestinal permeability of MSC-EVs was determined using an established model of the intestinal epithelium. Caco-2 epithelial cells were seeded at a density of 1 × 10^5^ cells/cm^2^ on Transwell cell culture inserts (polycarbonate semipermeable membrane, 1.12 cm^2^ area, 0.4 μm pore size, 0.5 mL in apical compartment and 1.5 mL in basolateral compartment; product #3401, Corning). The Caco-2 cells were maintained in culture for 19–21 days until they differentiated into enterocyte-like cells of the small intestine.^59^ The transepithelial electrical resistance (TEER) was measured every 2–3 days using an Epithelial Volt Ohm Meter (EVOM; World Precision Instruments) to assess the development of epithelial barrier integrity and formation of tight junctions in differentiated Caco-2 monolayers (Figure S4). The STX2/chopstick electrodes of the EVOM were sterilized in 70% ethanol and neutralized in DMEM prior to taking measurements. The resistance measurements were corrected for the blank and multiplied by the membrane area of the Transwell inserts (1.12 cm^2^) to obtain TEER values in ohm-centimeter squared (Ω·cm^2^). TEER values ≥ 500 Ω·cm^2^ are indicative of fully formed tight junctions in the Caco-2 monolayer.^60^

For the permeability assay, the culture medium was removed from differentiated Caco-2 cells in transwell and the cells were incubated with Hanks’ Balanced Salt Solution (HBSS; product #H8264, Sigma-Aldrich) at 37 °C for 1 h. The HBSS solution in the apical (upper) compartment was then removed and WGA-AF488-labeled EVs (1 × 10^9^ particles/mL) prepared in HBSS were applied to the Caco-2 monolayer and incubated at 37 °C for 3 h. Samples (100 μL) were collected from the basolateral (lower) compartment every 30 min and replaced with an equal volume of HBSS to maintain equilibrium. The particle concentration of transported EVs was measured using fluorescence intensity at Ex/Em 490/530 and nanoparticle tracking analysis (NTA). Hourly samples were also analyzed for particle size using NTA, and cryogenic electron microscopy (cryo-EM) was used to image transported vesicles. The TEER was measured before and after the permeability assay in HBSS to monitor changes in Caco-2 barrier integrity.

#### Cellular Uptake of MSC-EVs

Caco-2 epithelial cells were seeded at a density of 1 × 10^5^ cells/cm^2^ on Transwell inserts with polycarbonate membranes (product #3401, Corning) and maintained in culture for 19–21 days until differentiation. J774A.1 macrophages were seeded in 12-well plates (Thermo Fisher Scientific) at a density of 5 × 10^5^ cells/mL (1 mL/well). WGA-AF488-labeled EVs (1 × 10^9^ particles/mL) were applied to the cells and incubated at 37 °C for 4 h. Subsequently, differentiated Caco-2 cells were detached using TrypLE Express (1×) stable trypsin replacement enzyme (product #12605–010, Thermo Fisher Scientific) and J774A.1 macrophages were detached using a cell scraper and centrifuged at 1500 rpm for 10 min. The cells were then stained with DAPI in 2% bovine serum albumin (BSA) and analyzed with a Cytoflex LX flow cytometer (Beckman Coulter) at FITC channel to quantify EV uptake in differentiated Caco-2 intestinal epithelial cells (Figure S5a) and J774A.1 macrophages (Figure S6a). Unstained cells were used as a control to set the gating (Figure S7). At least 10,000 events were analyzed and data were processed using CytExpert 2.5 software (Beckman Coulter).

The uptake of MSC-EVs by differentiated Caco-2 intestinal epithelial cells was imaged using confocal microscopy (Figure S5b). Caco-2 cells were seeded at a density of 1 × 10^5^ cells/cm^2^ on Transwell inserts with polyester membranes (product #3460, Corning) and cultured for 19–21 days until differentiation. Differentiated Caco-2 cells were incubated with WGA-AF488-labeled EVs (1 × 10^10^ particles/mL) at 37 °C for 4 h. The cells were then washed with PBS and fixed with paraformaldehyde 4% fixative (product #30400006, bioWorld) for 20 min at room temperature. The cells were permeabilized with 0.2% (v/v) Triton X-100 (product #T8787, Sigma-Aldrich) in PBS for 5 min and blocked against nonspecific binding with blocking buffer (5% (w/v) skimmed milk powder and 0.05% (v/v) Triton X-100 in PBS) for 30 min at room temperature. Afterward, the cells were incubated overnight at 4 °C with rabbit anti-ZO-1 primary polyclonal antibody (product #40–2200, Thermo Fisher Scientific), diluted 1:150 in PBS containing 1% (w/v) skimmed milk powder and 0.5% (v/v) Triton X. After washing with PBS the next day, the cells were incubated for 1 h at room temperature, protected from light, with chicken antirabbit IgG (H+L) cross-adsorbed secondary antibody Alexa Fluor 647 (product #A21443, Thermo Fisher Scientific), diluted 1:500 in PBS containing 1% (w/v) skimmed milk powder and 0.05% (v/v) Triton X, to stain the tight junction protein zonula occludens (ZO)-1. Following another washing step, 1 drop of DAPI-containing mounting medium (Fluoroshield with DAPI; product #F6057, Sigma-Aldrich) was added to the inset and incubated for 10 min to stain the cell nuclei. The insert membrane was then excised using a scalpel and laid flat onto a microscopy μ-slide (product #80806, Ibidi), where it was immobilized using 0.5% (w/v) agarose gel in PBS (low gelling temperature agarose; product #A9414, Sigma-Aldrich). Fluorescence images were captured using a Nikon Eclipse Ti Inverted Spinning Disk confocal microscope with a Yokogawa CSU-X1 spinning disk unit and Andor EMMCD Camera. Images were processed using Fiji software.

The uptake of MSC-EVs by J774A.1 macrophages was imaged using imaging flow cytometry (Figure S6b). J774A.1 macrophages were seeded in 100 mm cell culture dishes (Thermo Fisher Scientific) at a density of 1 × 10^6^ cells/mL (5 mL/dish) and incubated with WGA-AF680-labeled EVs (1 × 10^9^ particles/mL) at 37 °C for 4 h. The cells were manually detached using a cell scraper, centrifuged at 1500 rpm for 10 min and resuspended in 0.5 mL PBS for analysis using the Cytek Amnis ImageStream Mark II imaging flow cytometer.

### MSC-EVs in a Coculture Model of Intestinal Inflammation

A Caco-2/J774A.1 coculture that mimics intestinal inflammation in vitro was established as described in detail.^44^ Caco-2 cells were seeded at a density of 1 × 10^5^ cells/cm^2^ on Transwell cell culture inserts (polycarbonate semipermeable membrane, 1.12 cm^2^ area, 0.4 μm pore size, 0.5 mL in apical compartment and 1.5 mL in basolateral compartment; product #3401, Corning). Caco-2 cells were maintained in culture for 19–21 days until they differentiated into polarized intestinal epithelial monolayers. Stabilization of transepithelial electrical resistance (TEER) values (∼2000 Ω·cm^2^) indicated the presence of an intact epithelial barrier with fully formed tight junctions. Differentiated Caco-2 cells were primed with an optimized cytokine cocktail of 25 ng/mL TNF-α, IFN-γ and IL-1β (product #GFH111, #GFH77 and #GFH167, respectively, Cell Guidance Systems) added to the basolateral compartment. On the same day, J774A.1 macrophages were seeded in 12-well plates (Corning) at a density of 5 × 10^4^ cells/well (1.5 mL/well) in culture medium containing 100 ng/mL LPS (product #L2630, Sigma-Aldrich) and 25 ng/mL IFN-γ. After 24 h, the two cell lines were combined into a coculture with primed Caco-2 epithelial cells in the apical compartment and activated J774A.1 macrophages in the basolateral compartment to mimic the inflamed intestine. MSC-EVs were then applied to the inflamed Caco-2/J774A.1 coculture either in the apical or basolateral compartment (final concentration 1 × 10^9^ particles/mL). The volumes in the apical and basolateral compartments of the Transwell cell culture system were maintained at equal level to prevent changes in osmolarity. Samples were collected from the basolateral compartment after 6 and 12 h to quantify pro-inflammatory cytokine (TNF-α and IL-6) production from macrophages using a Mouse Luminex Discovery assay (product #LXSAMSM, Bio-Techne). The TEER was measured at 12 and 16 h to monitor changes in the epithelial barrier integrity. TEER values were normalized to the baseline values of Caco-2 monocultures and expressed as percentages. A healthy coculture of differentiated Caco-2 cells and J774A.1 macrophages was used as a control.

### Stability of MSC-EVs in Gastrointestinal Fluids

MSC-EVs were incubated with dissolution media that simulate human gut fluids to mimic gastrointestinal digestion in vitro. EVs (∼2 × 10^9^ particles/mL) were first incubated with fasted state simulated gastric fluid (FaSSGF; pH 1.7), prepared according to the manufacturer’s instructions using FaSSGF buffer concentrate (product #FASGBUF, Biorelevant) and 3F Powder (product #FFF02, Biorelevant), in a 1:1 (v/v) ratio for 2 h at 37 °C with gentle orbital shaking. The resulting EV mixture was then incubated with fasted state simulated intestinal fluid (FaSSIF; pH 6.6), prepared according to the manufacturer’s instructions using FaSSIF buffer concentrate (product #FASBUF, Biorelevant) and 3F Powder, in a 1:1 (v/v) ratio for another 2 h at 37 °C with gentle orbital shaking. Digestive enzymes were also included following the static in vitro digestion protocol.^61^ Pepsin (product #P7012, Sigma-Aldrich) was added to simulated gastric fluid (GF) at a final concentration of 2000 U/mL and pancreatin (product #P1750, Sigma-Aldrich) was added to simulated intestinal fluid (IF) at a final concentration of 100 U/mL trypsin activity (based on TAME assay; 1 TAME unit = 19.2 USP units).^62^ Milli-Q water and 10% sodium dodecyl sulfate (SDS) were used as controls for EV disintegration.

Following incubation with GF and IF in the presence of digestive enzymes, EVs were filtered through Exo-spin columns (Exo-spin mini, product #EX03–50, Cell Guidance Systems) and analyzed using nanoparticle tracking analysis (NTA; Nanosight Pro NS500, Malvern Panalytical) to measure particle size distribution. MSC-EVs were also imaged using cryogenic electron microscopy (cryo-EM) 30 min and 1 h after digestion to capture vesicle morphology. EVs incubated with GF and IF (without digestive enzymes) were stained with anti-CD81 488 (1:100,000; product #CL488–65195, Proteintech) and anti-TSG101 647 (1:100,000; product #CL647–67381, Proteintech) and analyzed with a flow nanoanalyzer (NanoFCM) to determine particle concentration and percentage of EV subpopulations positive for transmembrane protein CD81 and/or cytosolic protein TSG101.

### Oral Formulation of MSC-EVs

#### Double-Coating Formulation and Characterization

MSC-EVs were sequentially coated with chitosan followed by Eudragit S-100 to form chitosan-Eudragit-coated EVs, referred to as coated EVs (Figure S8). A 0.1% (w/v) chitosan solution (product #448869, Sigma-Aldrich) was prepared in 0.5% (v/v) acetic acid (preadjusted to pH 5.5–5.7 with NaOH; product #A113–50, Fisher Scientific). The chitosan solution was stirred at 500 rpm for 1–2 h at room temperature, then stored at 4 °C. MSC-EVs in PBS (10^9^–10^10^ particles/mL) were slowly added dropwise to an equal volume of 0.1% chitosan solution under magnetic stirring (500 rpm) at room temperature. Chitosan-coated EVs were purified using a Float-A-Lyzer dialysis device (1 mL, 1000 kDa MWCO; product #G235037, Repligen) to remove excess chitosan. Dialysis was performed at 4 °C with the membrane submerged in 50 mM sodium acetate buffer (pH 5.5–5.7) in a 500 mL beaker for 6 h with gentle stirring (100 rpm), replacing the buffer every 2 h. The chitosan-coated EVs were then characterized for particle size and concentration by nanoparticle tracking analysis (NTA), surface charge by zeta potential, and morphology by cryogenic electron microscopy (cryo-EM). A 0.2% (w/v) Eudragit S-100 solution (Evonik) prepared in PBS (pH > 7) was slowly added dropwise to the chitosan-coated EVs under magnetic stirring (500 rpm) at room temperature. The appearance of white precipitates in the solution signaled the formation of a solid double-layer coating around the EVs. The addition of Eudragit was stopped when no further precipitates were observed and before the solution became visibly turbid due to excess polymer. The resulting chitosan-Eudragit-coated EVs were then washed several times with Milli-Q water (pH 6.5) by removing the supernatant and resuspending the precipitates in fresh water. A clear solution indicated removal of residual Eudragit not incorporated into the coating. The coated EVs were then stored at 4 °C until further use. Coated EVs were dried and imaged by scanning electron microscopy (SEM; JSM-6701F, JEOL).

#### Stability of Coated MSC-EVs in Gastrointestinal Fluids

Coated MSC-EVs were first incubated with FaSSGF for 2 h (1:1 volume ratio), followed by FaSSIF for another 2 h (1:1 volume ratio) at 37 °C with gentle orbital shaking. The supernatant of coated EVs was then stained with anti-CD81 488 (1:100,000) and anti-TSG101 647 (1:100,000) and analyzed with a flow nanoanalyzer (NanoFCM) to determine particle concentration of released EVs from the coating and percentage of CD81- and/or TSG101-positive EVs. Coated EVs were also incubated with FaSSGF containing pepsin (2000 U/mL) and FaSSIF containing pancreatin (100 U/mL trypsin activity) for 2 h at 37 °C with gentle orbital shaking. The coated EVs were then dried and visualized with scanning electron microscopy (SEM; JSM-6701F, JEOL). Uncoated EVs (∼2 × 10^9^ particles/mL) and coated EVs in water were used as controls.

#### MSC-EV Release from Double-Coating Formulation In Vitro

Coated MSC-EVs were incubated with fasted state simulated colonic fluid (FaSSCoF; pH 7.8), prepared according to the manufacturer’s instructions using FaSSCoF Powder (product #COFAS01, Biorelevant), containing chitosanase from *Streptomyces griseus* (final concentration 0.1 U/mL; product #C9830, Sigma-Aldrich) in a 1:1 (v/v) ratio for 6 h at 37 °C with gentle orbital shaking. The released EVs in the solution were filtered through Exo-spin columns (Exo-spin mini, product #EX03–50, Cell Guidance Systems) and analyzed with a flow nanoanalyzer (NanoFCM) to determine particle concentration and percentage of EVs expressing transmembrane protein CD81 and/or cytosolic protein TSG101. The released vesicles were also imaged using cryogenic electron microscopy (cryo-EM). Uncoated EVs (∼1 × 10^9^ particles/mL) were used as a control.

The biological activity of released EVs was determined by measuring TNF-α production in macrophages. J774A.1 macrophages were seeded in 96-well plates (Thermo Fisher Scientific) at a density of 2 × 10^5^ cells/mL (0.1 mL/well) in culture medium alone and in culture medium containing 100 ng/mL lipopolysaccharide (LPS) from *Escherichia coli* O111:B4 (product #L2630, Sigma-Aldrich) and 25 ng/mL interferon-γ (IFN-γ; product #GFH77, Cell Guidance Systems) to induce inflammation. The cells were incubated at 37 °C for 24 h. The next day, J774A.1 macrophages were treated with EVs released from the coating formulation (final concentration in wells: 10^9^ particles/mL) and incubated for a further 24 h. Uncoated MSC-EVs were used as a positive control for anti-inflammatory activity and cell culture medium was used as a negative control. TNF-α released from J774A.1 macrophages was quantified using a mouse Enzyme-Linked Immunosorbent Assay (ELISA; product #ELM-TNFa, RayBiotech), following the manufacturer’s protocol. The absorbance was measured at 450 nm using an Infinite 200 Pro plate reader (Tecan) and a standard curve was generated to quantify TNF-α levels in the samples.

### Coated MSC-EVs in a DSS-Induced Colitis Mouse Model

All animal experiments were approved by the Institutional Animal Care and Use Committee (IACUC) of Nanyang Technological University (NTU). C57BL/6J mice (male, 8–10 weeks old, body weight ≥ 20 g) were obtained from the NTU animal research facility and housed under standard conditions with a 12-h light/dark cycle and ad libitum access to food and water.

A total of 30 mice were randomly allocated to six groups (*n* = 5 per group). Mice in the healthy control group were housed together and given reverse osmosis (RO) water as standard drinking water for the duration of the study. Mice from the remaining five groups were cohoused to minimize cage effects. Acute colitis was induced by administering 3% (w/v) dextran sodium sulfate (DSS; 40 kDa MW, product #42867–100G, batch #BCCJ9094, Sigma-Aldrich) in drinking water ad libitum for five consecutive days, followed by administration of normal drinking water. Clinical signs of colitis, including weight loss, diarrhea, and occult blood in feces, were recorded daily and scored using an adapted scoring system (Table S1). Scores for each parameter were summed to calculate the disease activity index (DAI). Treatments were administered once daily during DSS exposure (days 1–5). Mice received MSC-EVs (1 × 10^9^ particles/mouse/day) via intravenous (IV) injection in the tail vein or by oral gavage (100 μL/dose). For oral delivery, either uncoated or coated EVs in RO water were administered. Healthy and DSS control groups received RO water orally (*n* = 3) or saline via IV injection (*n* = 2). An additional control group received coated empty liposomes in RO water orally. All preparations for in vivo administration were sterile-filtered or handled aseptically in a biosafety cabinet. Mice were euthanized by carbon dioxide inhalation and colons were excised and measured for length and weight in a blinded manner. Colons were also photographed on a flat surface and scale bars were generated in ImageJ from a ruler imaged in the same plane.

#### Biodistribution of MSC-EVs

MSC-EVs were labeled with wheat germ agglutinin conjugated to Alexa Fluor 680 (WGA-AF680; product #W32465, Thermo Fisher Scientific). Briefly, EVs were incubated with 0.1 mg/mL WGA-AF680 at 37 °C for 30 min under gentle agitation, protected from light. Labeled EVs were purified using Exo-spin columns (Exo-spin mini, product #EX03–50, Cell Guidance Systems). After labeling, the EVs were subjected to the coating process. Biodistribution studies were performed in two independent experiments (n = 2). Mice received 3% DSS in drinking water for 4 days to induce colitis and EVs (1 × 10^10^ particles/mouse) were administered by IV injection or oral gavage (uncoated and coated EVs). After 24 h, fluorescence images of excised colon, liver, spleen, heart, lungs, and kidneys were acquired using an in vivo imaging system (IVIS, Revvity) and analyzed with *Living Image* software (version 4.8.3) with background correction applied.

### Statistical Analysis

All results are expressed as mean ± SD. GraphPad Prism 11 was used to construct graphs and perform statistical analysis. A *p*-value <0.05 was considered statistically significant.

## Supplementary Material



## Data Availability

The data that support the findings of this study are available from the corresponding author upon reasonable request.

## Notes

Schematic illustration of MSC-EV isolation (Figure S1); MTS assay standard curve for Caco-2 cells (Figure S2); fluorescent labeling of MSC-EVs (Figure S3); TEER measurements in Caco-2 monolayers (Figure S4); uptake of MSC-EVs by differentiated Caco-2 cells (Figure S5) and J774A.1 macrophages (Figure S6); flow cytometry gating strategy for MSC-EV uptake (Figure S7); schematic illustration of the MSC-EV coating process (Figure S8); SEM images of coated MSC-EVs (Figure S9); disease activity scoring guide for DSS-induced colitis (Table S1); biodistribution of MSC-EVs in major organs of DSS-treated mice (Figure S10) (PDF)
