# Supplementary material for: Oral Delivery of Mesenchymal Stem Cell-Derived Extracellular Vesicles To Treat Intestinal Inflammation
Source: ACS Appl Mater Interfaces. 2026 Jun 30;18(28):38483–501. doi: 10.1021/acsami.6c07496 (PMC13422774; doi:10.1021/acsami.6c07496)
Supplement: Supplementary file 1 [file am6c07496_si_001.pdf]

Supporting Information

Oral Delivery of Mesenchymal Stem Cell-Derived Extracellular Vesicles to Treat Intestinal Inflammation

Mona Belaid [a b], Wei Heng Chng [b], Ram Pravin Kumar Muthuramalingam [b], Yun Wei Lim [c], Jana Javorovic [a], Yunyue Zhang [a], Xiang Luo [a], Bertrand Czarny [c]*, and Driton Vllasaliu [a]*

[a] *Institute of Pharmaceutical Science, King's College London, London SE1 9NH, United Kingdom*

[b] *Department of Pharmacy and Pharmaceutical Sciences, National University of Singapore, Singapore 117543, Singapore*

[c] *School of Materials Science and Engineering, Nanyang Technological University, Singapore 639798, Singapore*

**Corresponding authors: bczarny@ntu.edu.sg (B. Czarny); driton.vllasaliu@kcl.ac.uk (D. Vllasaliu)*

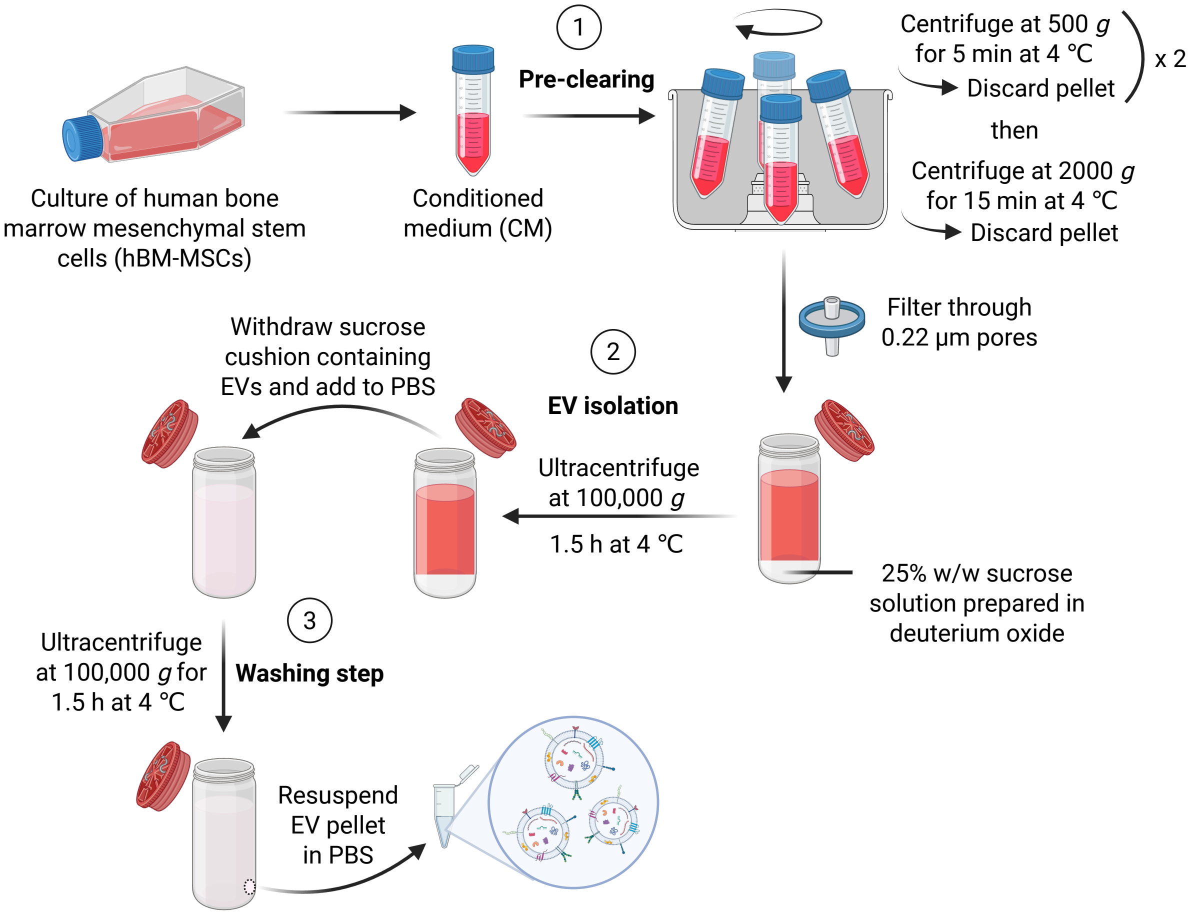

Figure S1. Schematic illustration of EV isolation from human bone marrow mesenchymal stem cells. The sucrose cushion ultracentrifugation technique is a density-based isolation method comprising three main steps: 1) pre-clearing of conditioned medium from dead cells and cell debris, 2) isolation of EVs onto a sucrose cushion and 3) washing step to remove sucrose and contaminating proteins. Created with BioRender.com.

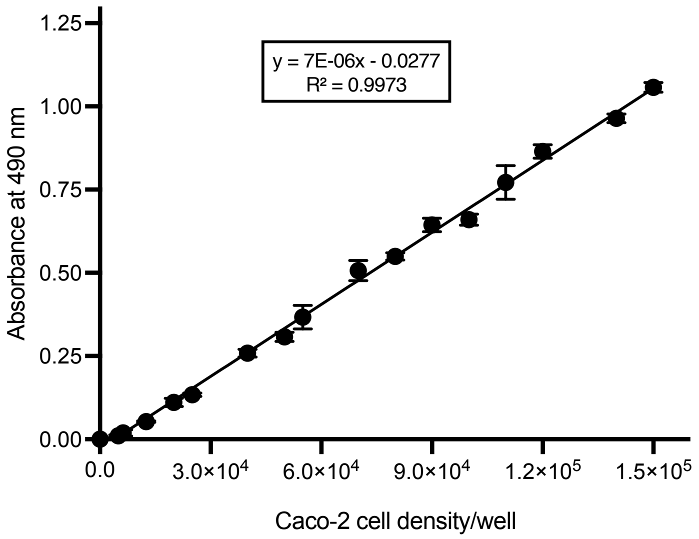

Figure S2. MTS assay standard curve for Caco-2 cells. Caco-2 cells were seeded at different densities in 48-well plates and incubated overnight at 37 °C to allow cells to adhere to the wells. The next day, the wells were washed with PBS before 200 µL of serum-free medium and 20 µL of MTS reagent were added to the wells. After 2 h incubation at 37 °C, the absorbance of the wells at 490 nm was recorded using a plate reader (Infinite 200 Pro, Tecan). Absorbance of the formazan product at 490 nm is proportional to Caco-2 cell density/well. Each data point represents a mean of 3 replicates ± SD.

a

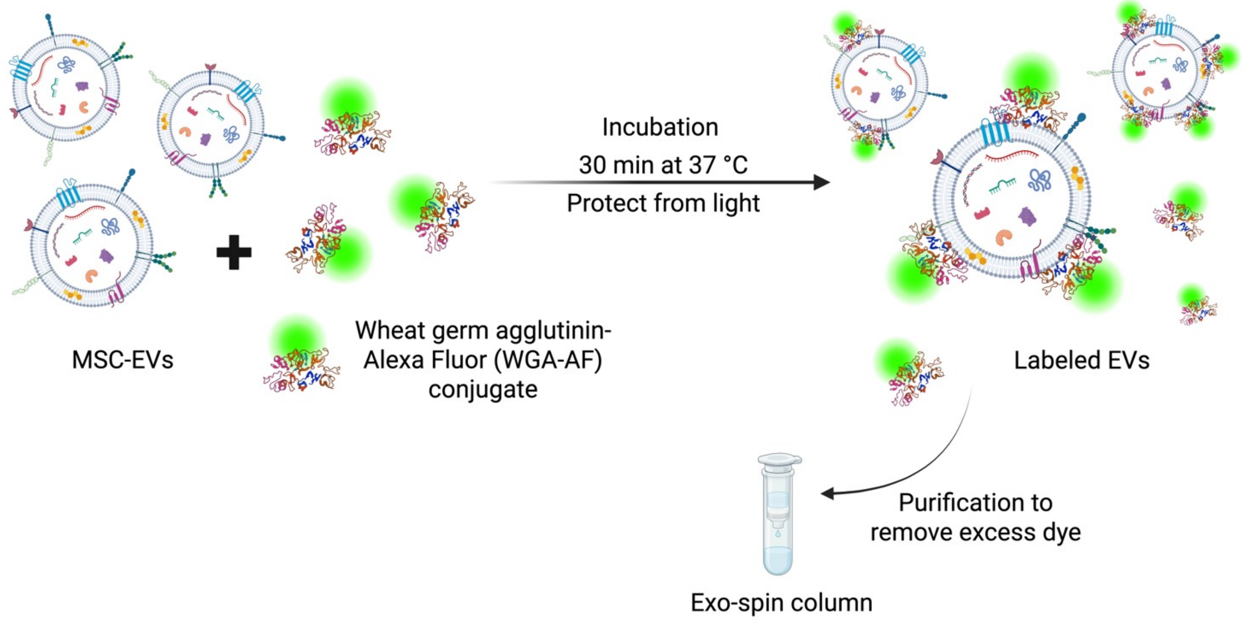

b

	EVs in PBS (before labeling)	Labeled EVs (after purification)	Dye alone (WGA-AF488)
Particle concentration (p/mL)	5.17×10^8 $\pm 3.71 \times 10^7$	4.79×10^8 $\pm 1.67 \times 10^7$	-
Mean particle size (nm)	147.7 ± 5.5	153.3 ± 15.1	-
Fluorescence intensity (Ex/Em: 490/530)	55	813	9281

Figure S3. Labeling of MSC-EVs with wheat germ agglutinin-Alexa Fluor (WGA-AF) conjugates. (a) MSC-EVs were incubated with WGA-AF488 or WGA-AF680 for 30 min at 37 °C with gentle agitation, protected from light. Excess dye was removed using Exo-spin columns (Cell Guidance Systems) to purify the EVs. **(b)** Characterization of MSC-EVs before and after fluorescent labeling. 90% of the EVs were recovered after labeling and purification, with no significant increase in the mean particle size. The size distribution and particle concentration were measured using nanoparticle tracking analysis (NTA) and the fluorescence intensity was measured using a plate reader (BioTek Synergy H1, Agilent).

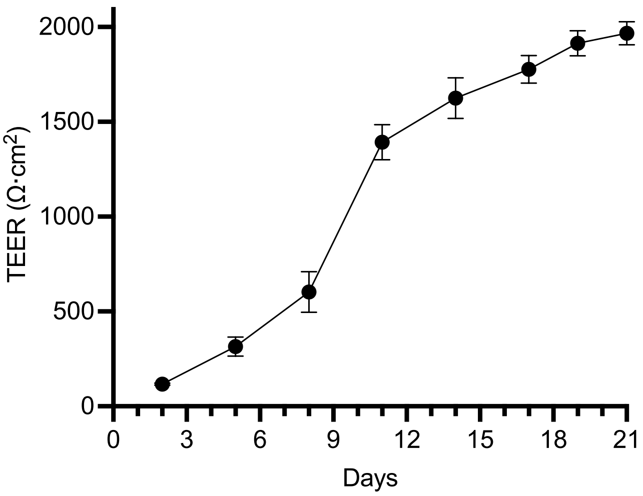

Figure S4. Transepithelial electrical resistance (TEER) measurements of Caco-2 monolayers over 21 days. Caco-2 epithelial cells were seeded on Transwell cell culture inserts and maintained in culture for 19-21 days to allow differentiation and stabilization of TEER values. TEER was measured every 2-3 days using an Epithelial Volt Ohm Meter (EVOM) to assess the development of barrier integrity and formation of tight junctions in differentiated Caco-2 monolayers. Each data point represents a mean of 5 independent replicates ± SD.

a

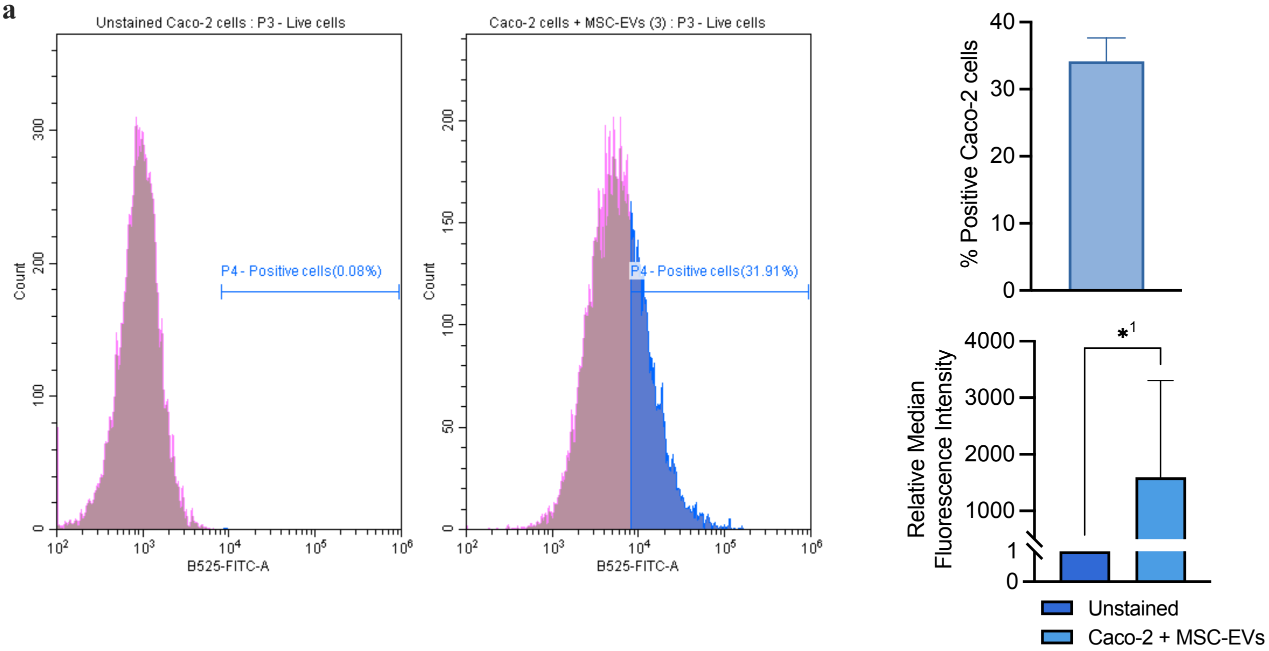

b

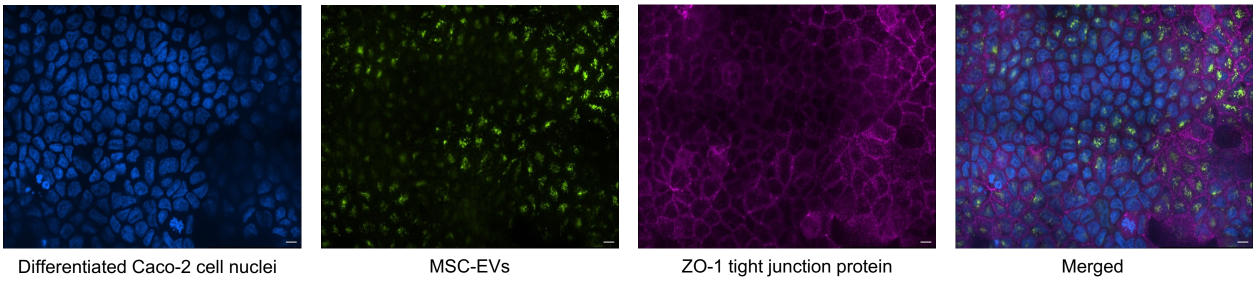

Figure S5. Uptake of MSC-EVs by differentiated Caco-2 epithelial cells. Differentiated Caco-2 cells were incubated with MSC-EVs labeled with WGA-AF488. After 4 h, the cells were analyzed using flow cytometry (Cytoflex LX, Beckman Coulter). **(a)** 35% of Caco-2 cells were positive for MSC-EVs and showed a significant increase in median fluorescence intensity (*[1] $p = 0.0453$). Data are presented as mean ± SD ($n = 2$) with t-test (* $p < 0.05$). **(b)** Fluorescence images of MSC-EVs in intestinal epithelial cells. EVs were labeled with WGA-AF488. Differentiated Caco-2 cell nuclei and tight junction protein ZO-1 were stained with DAPI and Alexa Fluor 647, respectively. Images were captured using a Nikon Eclipse Ti Inverted Spinning Disk confocal microscope. Scale bar: 20 µm.

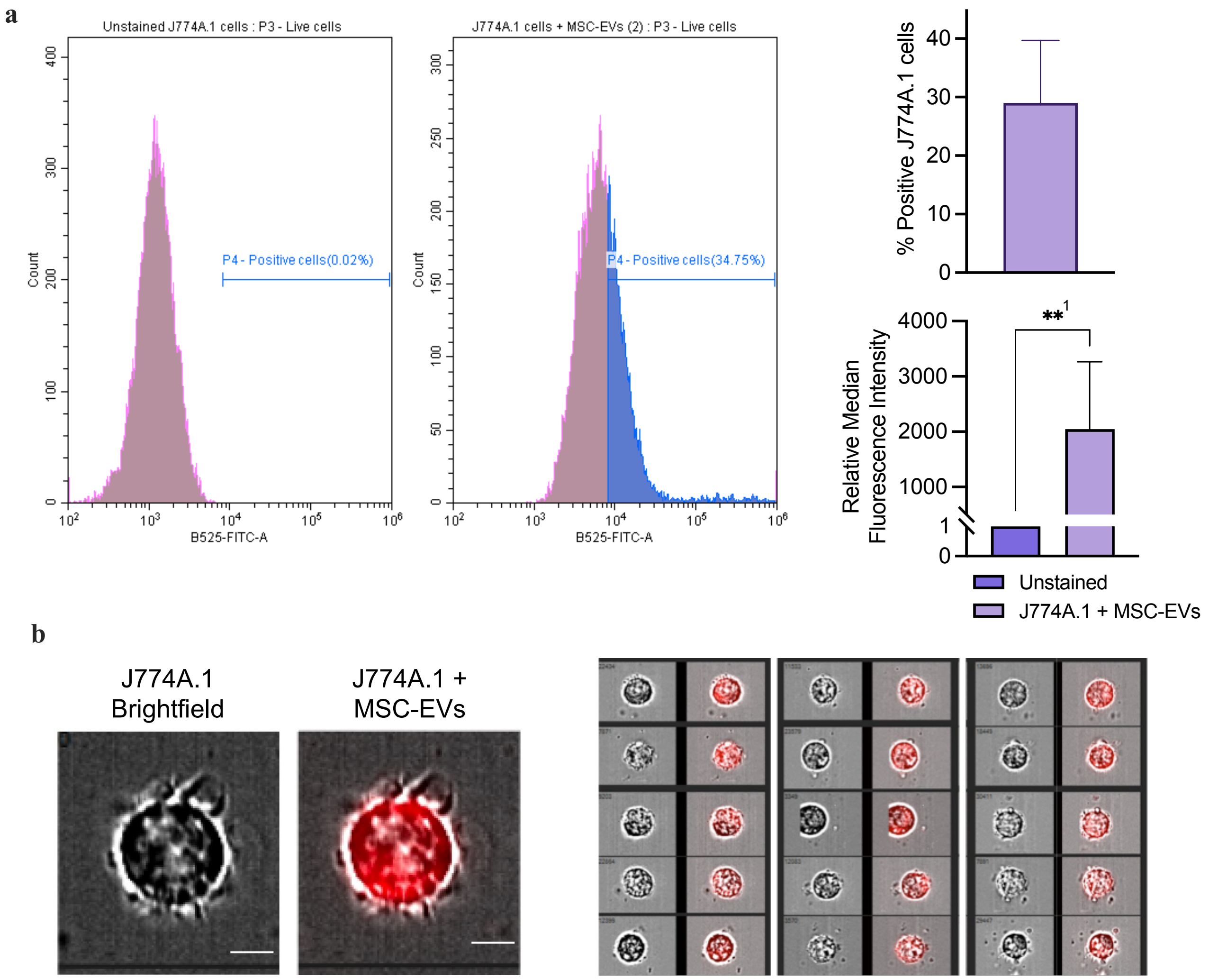

Figure S6. Uptake of MSC-EVs by J774A.1 macrophages. J774A.1 macrophages were incubated with MSC-EVs labeled with WGA-AF488. After 4 h, the cells were analyzed using flow cytometry (Cytoflex LX, Beckman Coulter). **(a)** 30% of J774A.1 cells were positive for MSC-EVs and showed a significant increase in median fluorescence intensity (**[1] $p = 0.0021$). Data are presented as mean ± SD ($n = 2$) with t-test (** $p < 0.01$). **(b)** Fluorescence images of MSC-EVs in J774A.1 macrophages. EVs were labeled with WGA-AF680. Images were captured using a Cytek Amnis ImageStream Mark II imaging flow cytometer. Scale bar: 5 µm.

a

(i)

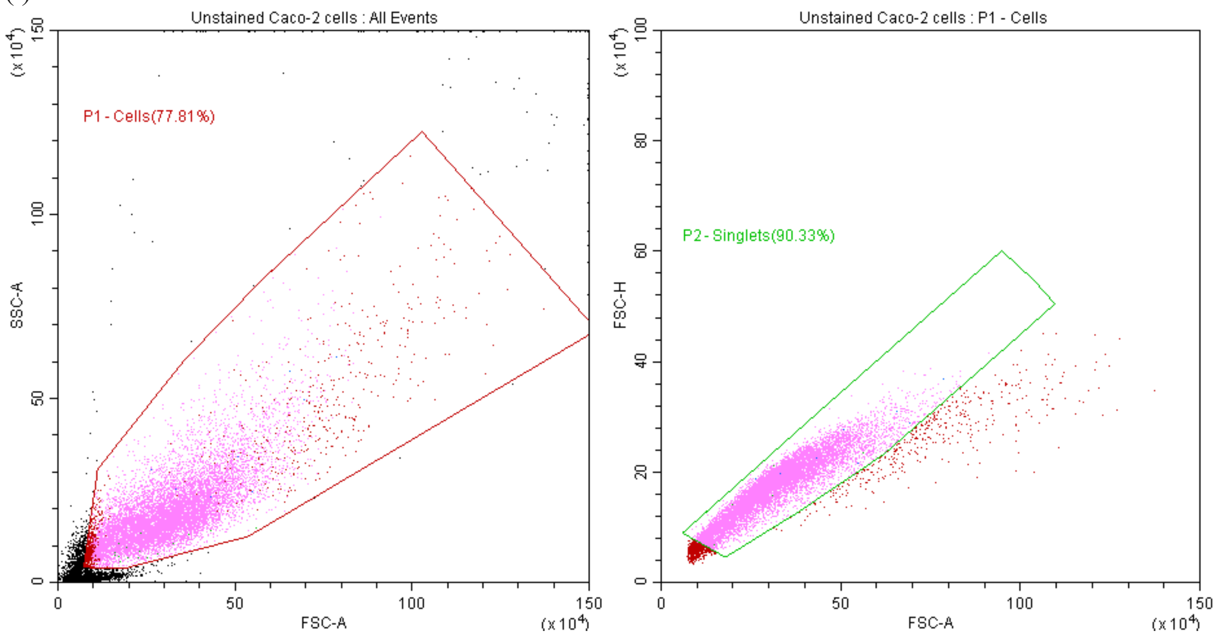
Unstained Caco-2 cells : All Events
P1 - Cells(77.81%)
SSC-A
FSC-A
Unstained Caco-2 cells : P1 - Cells
P2 - Singlets(90.33%)
FSC-H
FSC-A

(ii)

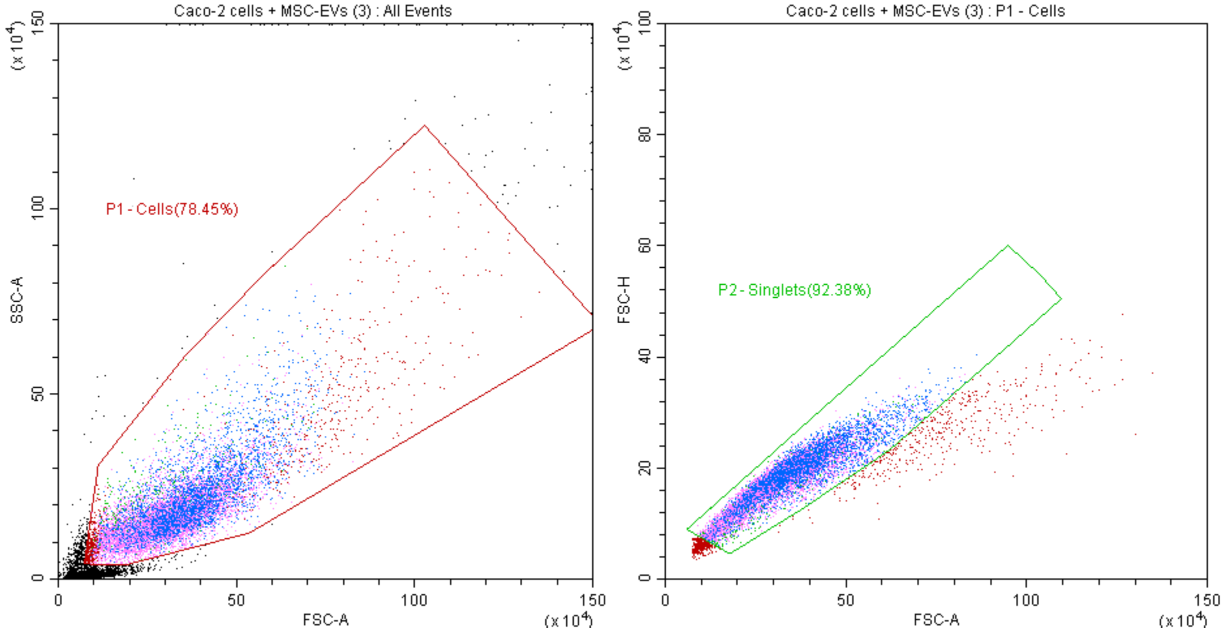
Caco-2 cells + MSC-EVs (3) : All Events
P1 - Cells(78.45%)
SSC-A
FSC-A
Caco-2 cells + MSC-EVs (3) : P1 - Cells
P2 - Singlets(92.38%)
FSC-H
FSC-A

b (i)

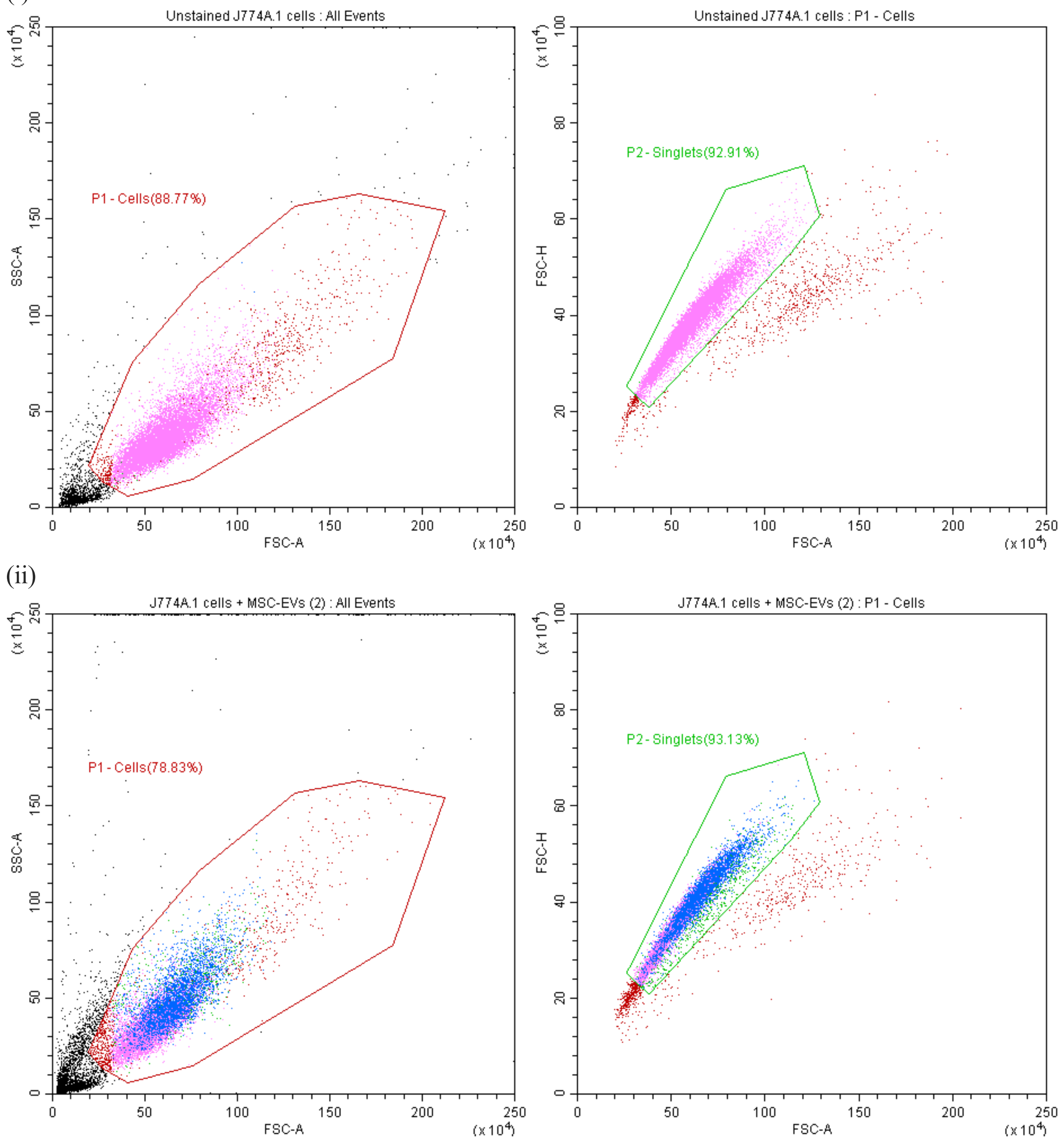

Figure S7. Flow cytometry gating strategy for MSC-EV uptake analysis. Gating was manually set using CytExpert software (Beckman Coulter) to assess MSC-EV uptake in **(a)** differentiated Caco-2 epithelial cells and **(b)** J774A.1 macrophages. Representative plots show (i) unstained cells and (ii) positive cells.

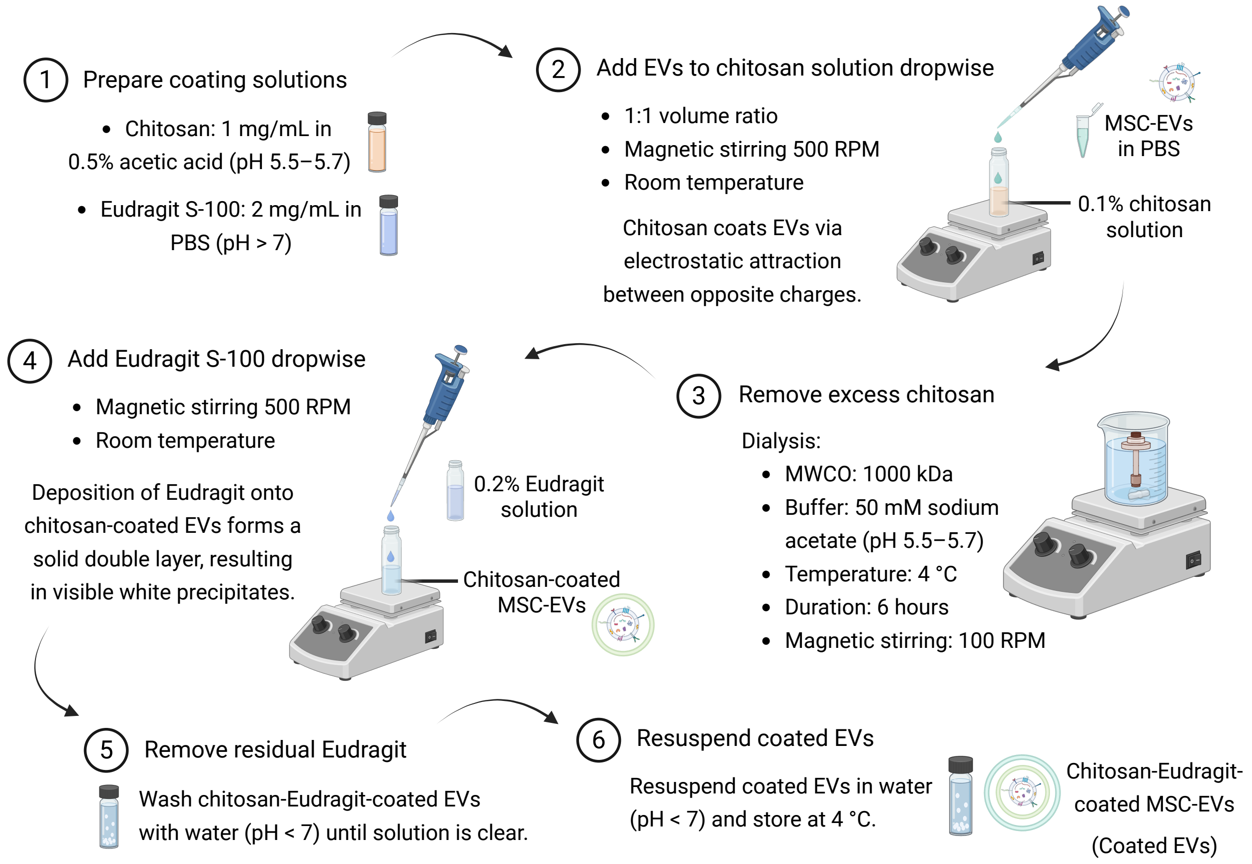

Figure S8. Schematic illustration of the MSC-EV coating process. MSC-EVs were sequentially coated with chitosan as the first layer and Eudragit S-100 as the second layer, resulting in chitosan-Eudragit-coated EVs (referred to as coated EVs). Created with BioRender.com.

a

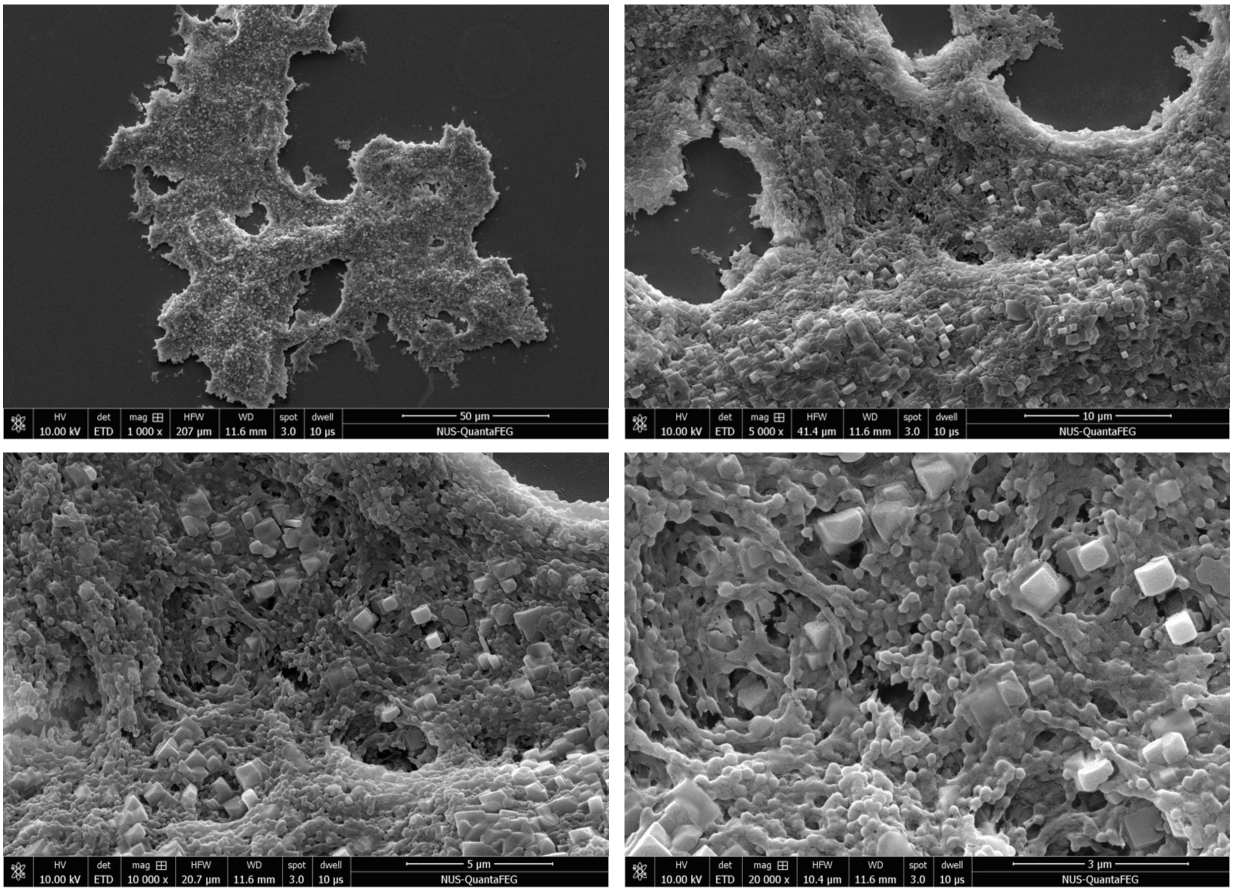
HV det mag HFW WD spot dwell
10.00 kV ETD 1 000 x 207 µm 11.6 mm 3.0 10 µs
50 µm
NUS-QuantaFEG
HV det mag HFW WD spot dwell
10.00 kV ETD 5 000 x 41.4 µm 11.6 mm 3.0 10 µs
10 µm
NUS-QuantaFEG
HV det mag HFW WD spot dwell
10.00 kV ETD 10 000 x 20.7 µm 11.6 mm 3.0 10 µs
5 µm
NUS-QuantaFEG
HV det mag HFW WD spot dwell
10.00 kV ETD 20 000 x 10.4 µm 11.6 mm 3.0 10 µs
3 µm
NUS-QuantaFEG

b

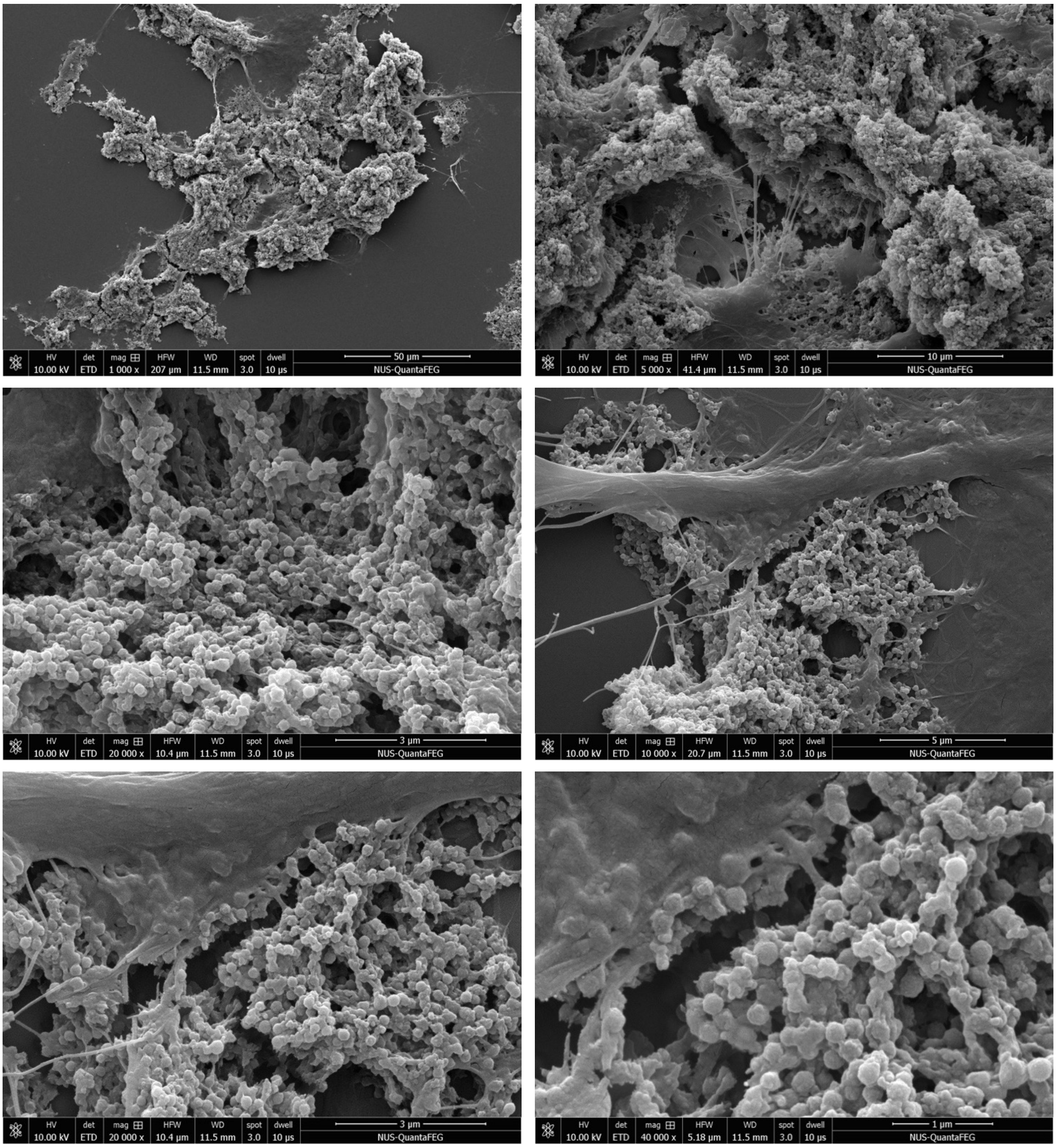

Figure S9. Scanning electron microscopy (SEM) images of coated MSC-EVs (a) in water (pH 6.5) and **(b)** after incubation with simulated gastric and intestinal fluids and digestive enzymes (pepsin and pancreatin). Images of coated EVs revealed spherical particles embedded within a surrounding matrix structure, which was preserved following exposure to simulated gastrointestinal fluids and digestive enzymes. Images were acquired using a JEOL JSM-6701F field emission scanning electron microscope.

Score	Weight loss	Stool consistency		Fecal occult blood	
0	<1%	Hard and dry	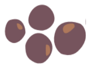	No visible blood and minimal on screen	
1	1-5%	Slightly softer	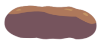	No visible blood but noticeable on screen (light blue color)	
2	5-10%	Soft and smooth	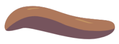	Slightly noticeable (slight maroon color) but evident on screen (blue color)	
3	10-15%	Very soft but still maintains a shape	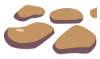	Noticeable (strong maroon color) and very evident on screen (dark blue color)	
4	>15%	Diarrhea	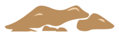	Blood is visually obvious without a screen test	

Table S1. Adapted disease activity scoring guide for body weight loss, stool consistency and fecal occult blood in mice with DSS-induced colitis. Blood presence in stools was assessed using the Hema-Screen test. Scores for each parameter were summed to calculate the disease activity index (DAI).

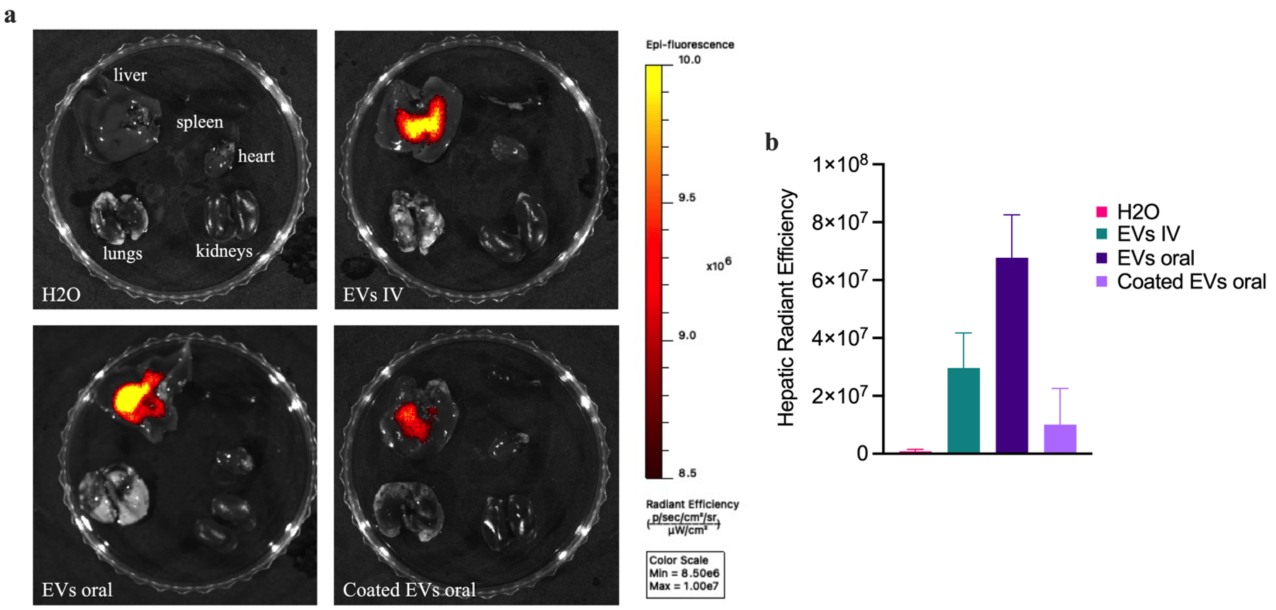

Figure S10. Biodistribution of MSC-EVs in major organs of DSS-treated mice. (a) Biodistribution of MSC-EVs in the liver, spleen, heart, lungs and kidneys of dextran sodium sulfate (DSS)-treated mice 24 h after EV administration. WGA-AF680-labeled EVs were administered via IV injection and oral gavage for uncoated and coated EVs, and the fluorescence in excised organs was imaged using an in vivo imaging system (IVIS, Revvity). **(b)** Quantification of EV fluorescence signal (radiant efficiency) in the liver 24 h after administration. Coating of EVs reduced hepatic fluorescence intensity compared with uncoated EVs administered orally or intravenously. Data are presented as mean ± SD from two independent experiments (n = 2).
